# Ni nanoparticles on RGO as reusable heterogeneous catalyst: effect of Ni particle size and intermediate composite structures in C–S cross-coupling reaction

**DOI:** 10.3762/bjoc.13.174

**Published:** 2017-08-28

**Authors:** Debasish Sengupta, Koushik Bhowmik, Goutam De, Basudeb Basu

**Affiliations:** 1 Department of Chemistry, University of North Bengal, Darjeeling 734013, India. Fax: +91 353 2699001; Tel: +91 353 2776381; 2 Nano-Structured Materials Division, CSIR–Central Glass & Ceramic Research Institute, 196, Raja S. C. Mullick Road, Jadavpur, Kolkata 700032, India. Fax: +91 33 24730957; Tel: +91 33 23223403

**Keywords:** C–S cross-coupling, heterogeneous catalyst, Ni nanoparticle, reduced graphene oxide, thioether

## Abstract

The present work demonstrates the C–S cross-coupling reaction between aryl halides and thiols using nickel nanoparticles (Ni NPs) supported on reduced graphene oxide (Ni/RGO) as a heterogeneous catalyst. It is observed that the uniformly dispersed Ni NPs supported on RGO could exhibit excellent catalytic activity in C–S cross-coupling reactions and the catalytic application is generalized with diverse coupling partners. Although the electron-rich planar RGO surface helps in stabilizing the agglomeration-free Ni NPs, the catalytic process is found to occur involving Ni(II) species and the recovered catalyst containing both Ni(0)/Ni(II) species is equally efficient in recycle runs. A correlation of loading of Ni species, size of NPs and the intermediate Ni-related heterostructures formed during the catalytic process has been established for the first time, and found to be best in the C–S cross-coupling reaction for Ni(0) and Ni(II) NPs of the average sizes 11–12 nm and 4 nm, respectively.

## Introduction

The formation of a carbon–sulfur bond is an imperative step for the synthesis of many biologically active chemical entities that have significant applications in different therapeutic areas such as HIV, cancer, diabetes, inflammation, Alzheimer’s and Parkinson’s diseases etc. [1–4]. The first palladium-catalyzed arylation of thiols was reported by Migita and co-workers in 1980 [5], and soon after Cristau and co-workers developed a nickel-catalyzed route for C–S cross-coupling reactions [6]. Other metals such as copper [7], cobalt [8], iron [9], rhodium [10], manganese [11], indium [12], and bismuth [13] have been used with specific electron-rich ligands in the C–S coupling reactions. However, these are less common compared to other C–X (X = C, O, N, P) coupling reactions, presumably because sulfur might suppress the catalytic function through its coordinating and adsorptive properties [14]. The Ni-catalyzed C–S cross-coupling reactions generally involved Ni salts [15–18], Ni–phosphine complexes [19–21], or Ni–NHC complexes [22–23], although the actual catalyst is believed to be a Ni(0)/Ni(I) species [16–21]. NiO supported on zirconia (NiO–ZrO_2_) is thus far known to act as the only heterogeneous nanocatalyst for C–S cross-coupling with limited applications and poor yields [24].

In recent years, graphene-based composite materials have become popular because of their unique physical, mechanical and chemical properties [25–27]. Graphene, a single atomic layer of conjugated sp^2^ carbon atoms with a large contact area, can adopt several guest particles [28–29]. Reduced graphene oxide (RGO) with a high surface area can be easily dispersed in aqueous or non-aqueous media and can be mingled with other nanomaterials to produce stable nanocomposites [30]. Therefore, RGO is considered an excellent candidate for catalyst support [31–32]. To date, various magnetic or semiconducting nanoparticles (NPs) have been incorporated in GO surfaces and thoroughly studied in terms of their photocatalytic and electrochemical properties [33–37]. However, only few graphene-based metal nanocomposites have been recognized for organic cross-coupling reactions [38–42]. In general, bare Ni NPs are very unstable and readily oxidized in air [43]. A few literature reports are available where Ni species immobilized on a solid surface have been used in C−C or C−N cross-coupling reactions [44–47]. We have recently shown that uniformly dispersed Ni NPs that are free of agglomeration can be embedded in RGO sheets (Ni/RGO). This very stable Ni/RGO nanocomposite enhances the reduction rate of Cr(VI) species to Cr(III) in the presence of formic acid at room temperature [48]. Also, it can serve as an excellent catalyst for the Kumada–Corriu C–C cross-coupling reaction [49]. Since heterogeneous Ni catalysts are rarely studied for the C–S cross-coupling reaction between aryl halides and thiols, presumably because of the fact that the thiols (bearing -S–H) might poison the catalyst, we became interested to examine this catalyst. Herein, we present our studies which revealed that both Ni(0) and Ni(II) species formed during the reaction and remaining on RGO could act as the active catalysts for the C–S cross-coupling reaction. Moreover, a correlation between the loading of metal, the effect of average NP size of Ni(0)/Ni(II) species in the catalyst and the intermediate composite heterostructures has been established for the first time.

## Results and Discussion

We synthesized Ni/RGO nanocomposites following our reported method [48–49]. These materials were well characterized by powder XRD, TEM, TGA and XPS studies. The preparation and characterization of the nanocomposites were described in detail in our previous publications [48–49]. Usually, bare Ni NPs are unstable and likely to undergo aerial oxidation [43]. However, in the Ni/RGO composite, the electron-rich RGO surface helps in stabilizing the Ni in its zero-valent state. The high surface area and planar structure of RGO prevent the Ni NPs (to an optimum loading of Ni) from agglomeration and provide enough adsorption sites to the reacting molecules. In this work graphene oxide (GO) was used as a support for in situ growth of Ni nanoparticles. The synthetic protocol presumably allows initial interaction between the metal and functional groups on to the basal plane of GO [48]. The interaction could take place effectively due to the planar structure and large contact area of GO. As a result, when reduced, the Ni nanoparticles could sit on the RGO surface without agglomeration.

We prepared three different Ni/RGO nanocomposites by varying the loading of Ni NPs. The crystallite sizes of Ni NPs in these three Ni(0)/RGO samples were calculated with X-ray line broadening using the (111) and (200) peaks as reported [48–49] (see also Supporting Information File 1). The average crystallite sizes of Ni NPs are 10, 11 and 17 nm in Ni/RGO-20 (20 wt % Ni), Ni/RGO-40 (40 wt % Ni) and Ni/RGO-60 (60 wt % Ni) samples, respectively. It was observed that an optimum loading of 40 wt % Ni in Ni/RGO could be loaded without any agglomeration with a restriction of average size ≈11 nm. However, if the loading is increased to 60 wt %, the average size of NPs becomes significantly higher (17 nm) due to agglomeration. Therefore, we found Ni/RGO-40 (40 wt % Ni) is very suitable for our catalytic reactions.

We began our studies on optimizing the reaction taking 3-iodoanisole and benzenethiol as model coupling partners and Ni/RGO as the catalyst. In the process, we first examined the coupling reactions with Ni/RGO-40 at varying temperatures, bases and solvents, though other nanocomposites (Ni/RGO-20 and Ni/RGO-60) were also tested (Table 1). All reactions were carried out under a N_2_ atmosphere to avoid oxidative dimerization of thiols to disulfide [50]. Solvent optimization was started with water (Table 1, entry 1) followed by toluene (Table 1, entry 2) and isolating diaryl sulfide only 6–8% after 10 h, while in DMSO the yield significantly rises to 86% within 3 h (Table 1, entry 3). The conversion was even higher (92%) when DMSO was replaced with DMF (Table 1, entry 4). However, a drop in the catalyst loading or a lowering of the temperature affected the course of the reaction by suppressing the overall yield of the thioether (Table 1, entries 5 and 6). Without using any base, the yield was 74%, while the use of KOH as a base afforded the thioether in 83% yield (Table 1, entries 7 and 8). Formation of the diphenyl disulfide via oxidative dimerization of benzenethiol was noticed (15%) when the reaction was carried out under aerobic conditions (Table 1, entry 9). Control experiments without the catalyst (Ni/RGO-40) or only with RGO (Ni-free) did not produce any cross-coupled sulfane product (Table 1, entries 10 and 11). The reaction was then performed in the presence of Ni/RGO-20 and Ni/RGO-60 under similar conditions affording the thioether in 91% and 84% yields, respectively (Table 1, entries 12 and 13). These observations suggest that both Ni/RGO-20 and Ni/RGO-40 with average Ni NPs size (≈10–11 nm) have better catalytic efficiency (yield 91–92%) than Ni/RGO-60 (≈17 nm), presumably attributable to the larger active surface areas in the former two cases. On the other hand, bare Ni NPs (of an average size ≈10 nm) prepared following the reported procedure [51], gave the corresponding cross-coupled product in 79% yield (Table 1, entry 14). This could be due to the agglomeration of NPs or further oxidation of Ni NPs in the absence of the electron-rich RGO surface.

**Table 1:** Optimization of the C–S cross-coupling reaction conditions using Ni/RGO-40.^a^

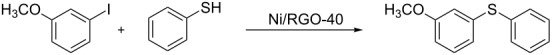						

Entry	Catalyst Ni content (mol %)	Solvent	Base	Temperature (°C)	Time (h)	Yield^b^ (%)

1	15	water	K_2_CO_3_	100	10	8
2	15	toluene	K_2_CO_3_	100	10	6
3	15	DMSO	K_2_CO_3_	100	3	86
**4**	**15**	**DMF**	**K_2_CO_3_**	**100**	**3**	**92**
5	10	DMF	K_2_CO_3_	100	3	81
6	15	DMF	K_2_CO_3_	80	10	61
7	15	DMF	None	100	10	74
8	15	DMF	KOH	100	3	83
9^c^	15	DMF	K_2_CO_3_	100	3	63
10	none	DMF	K_2_CO_3_	100	10	0
11^d^	RGO	DMF	K_2_CO_3_	100	10	0
12^e^	15	DMF	K_2_CO_3_	100	3	91
13^f^	15	DMF	K_2_CO_3_	100	3	84
14^g^	15	DMF	K_2_CO_3_	100	3	79
15^h^	15	DMF	K_2_CO_3_	100	3	92

^a^3-Iodoanisole (1 mmol), benzenethiol (1.2 mmol), K_2_CO_3_ (1.2 mmol) and solvent (3 mL) heated at 100 °C under N_2_. ^b^Isolated yield. ^c^Reaction was performed without N_2_. ^c^Diphenyl disulfide was isolated (15%). ^d^RGO (13.2 mg). ^e^Ni/RGO-20 catalyst. ^f^Ni/RGO-60 catalyst. ^g^Ni NPs. ^h^4-Iodoanisole (1 g, 4.27 mmol), benzenethiol (5.12 mmol), K_2_CO_3_ (5.12 mmol), Ni/RGO-40 catalyst (94.0 mg; Ni content is 37.60 mg, 0.64 mmol) and solvent (4 mL) heated at 100 °C under N_2_.

Thus, the average size of Ni NPs supported with electron-rich RGO surface seems to be important to obtain maximum catalytic efficiency in the C–S coupling reaction. The catalytic reaction was found to be scalable with comparable conversions (Table 1, entry 15).

We then extended the optimized reaction conditions (as in Table 1, entry 4) using Ni/RGO-40 to diverse functionalized aryl halide/thiol combinations. The results are indeed encouraging and summarized in Table 2. Both coupling partners, i.e., the iodoarenes and arylthiols bearing different groups like Me, OMe, F, COCH_3_, NO_2_ are equally efficient to undergo cross-coupling reactions producing the corresponding unsymmetrical diaryl sulfides **3a–i** in 88–93% isolated yields (Table 2, entries 1–9). No significant influence of electron-donating or electron-withdrawing groups has been noticed, which is in agreement with previously reported results in C–S coupling reactions [19]. In the case of iodobromoarenes or iodochloroarenes, we obtained only iodo-coupled products **3j** and **3k** in excellent yields (Table 2, entries 10 and 11). Bromoarenes remain unchanged under the given conditions. However, the cross-coupling of bromoarenes could be performed efficiently in the presence of zinc dust (1 equivalent to the substrate bromoarene) in addition to a catalytic amount of Ni/RGO-40 (Table 2, entries 12 and 13). Although the exact role of zinc is not clearly understood, based on literature reports [7,16], we presume that the oxidized organonickel intermediate Ar–Ni(II)–Br (derived from the oxidative addition of Ni to the Ar–Br bond) might not be sufficiently reactive to the thiolate anion (Ar–S^−^K^+^) before being reduced to the organonickel species [Ar–Ni(I)] in the presence of zinc. Diiodobenzenes, however, underwent smooth coupling affording the bis-coupled products **3m** and **3n** as the single products (Table 2, entries 14 and 15). Using aliphatic thiols has also resulted in the formation of unsymmetrical aryl alkyl thioether **3o** and **3p** in relatively lower yields (75–80%; Table 2, entries 16 and 17). Aliphatic thiols are, however, known to be less reactive as compared to aromatic thiols in coupling chemistry, which might be the reason to obtain lower yields [15].

**Table 2:** Ni/RGO-40 catalyzed C–S cross-coupling between aryl halide and thiol.^a^

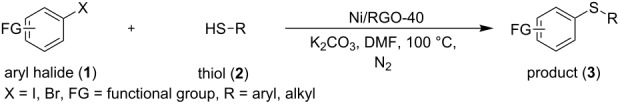					

Entry	Aryl halide (**1**)	Thiol (**2**)	Time (h)	Product (**3**)	Yield^b^ (%)

1	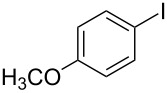	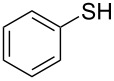	2	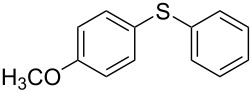 **3a**	93
2	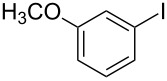	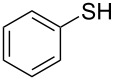	3	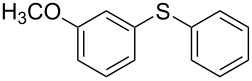 **3b**	92
3	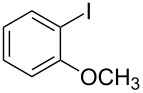	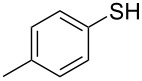	5	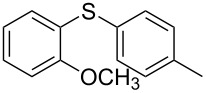 **3c**	90
4	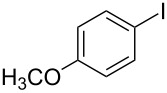	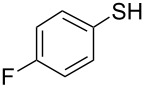	4	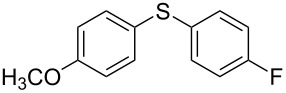 **3d**	91
5	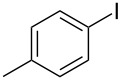	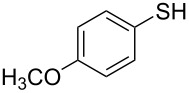	3	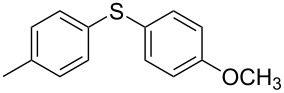 **3e**	93
6	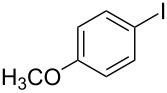	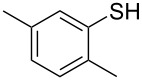	4	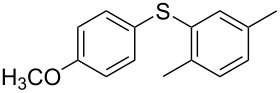 **3f**	90
7	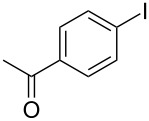	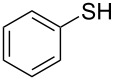	8	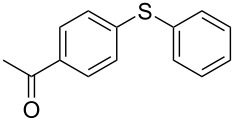 **3g**	88
8	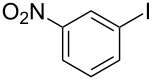	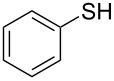	8	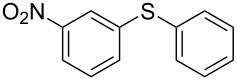 **3h**	90
9	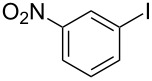	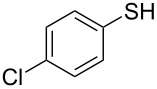	8	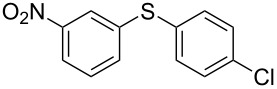 **3i**	89
10	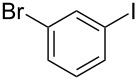	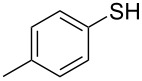	6	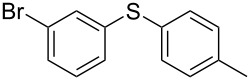 **3j**	90
11	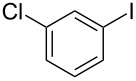	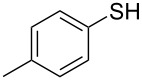	6	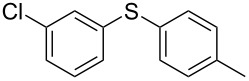 **3k**	88
12^c^	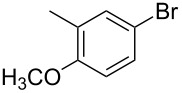	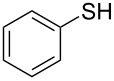	10	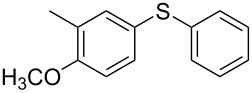 **3l**	85
13^c^	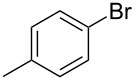	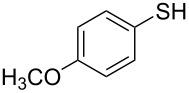	10	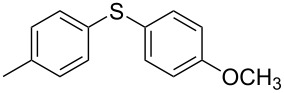 **3e**	88
14^d^	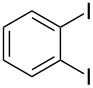	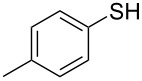	8	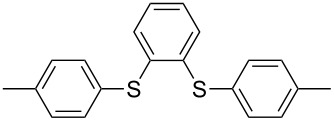 **3m**	87
15^d^	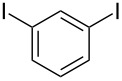	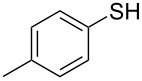	8	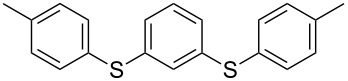 **3n**	84
16	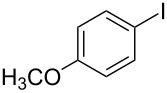	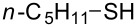	10	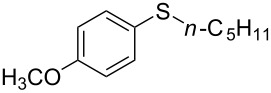 **3o**	80
17	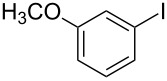	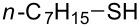	10	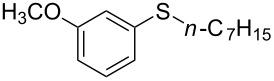 **3p**	75

^a^Aryl halide (1 mmol), thiol (1.2 mmol), K_2_CO_3_ (1.2 mmol), Ni/RGO-40 (40 wt %, 22 mg) and DMF (3 mL) heated at 100 °C under nitrogen. ^b^Isolated yield. ^c^Zn dust (1 mmol) was added. ^d^Aryl halide (0.5 mmol), 4-methylphenylthiol (1.2 mmol), K_2_CO_3_ (1.2 mmol), Ni/RGO-40 (40 wt %, 22 mg).

### Reusability of the catalyst (Ni/RGO-40)

**Figure 1:**
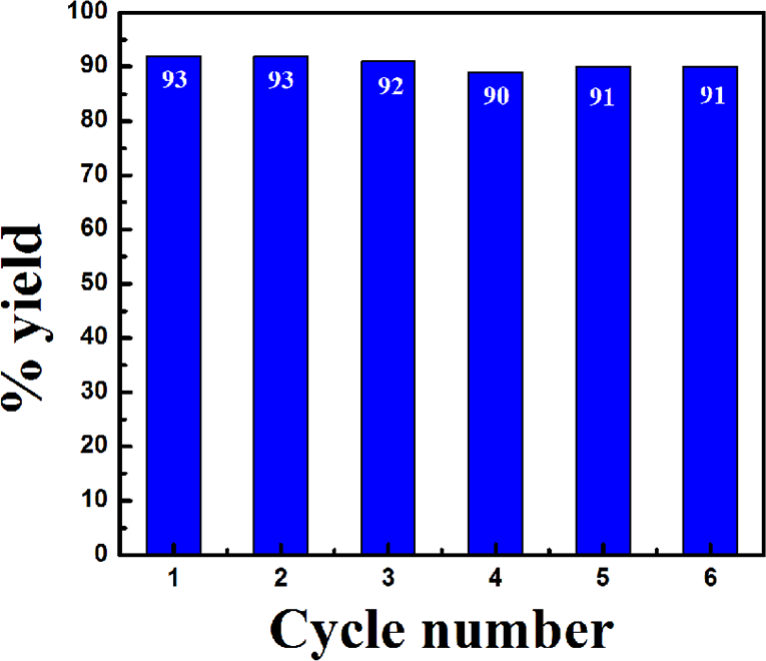
Recycling experiments of Ni/RGO-40 catalyst in C−S cross-coupling reaction between 4-iodoanisole and benzenethiol.

After establishing the generality of RGO-supported Ni NPs size-specific catalytic efficiency in the C–S coupling reaction, we recovered the catalyst from the reaction mixture (see Experimental section) and studied its reusability. Here, we used 4-iodoanisole and benzenethiol as the model coupling partners. It was found that the catalyst can be reused at least for six times for the same reaction examined consecutively without any significant drop in catalytic activity. Figure 1 shows the results of consecutive recycle runs.

### Characterization of the recovered catalyst

**Figure 2:**
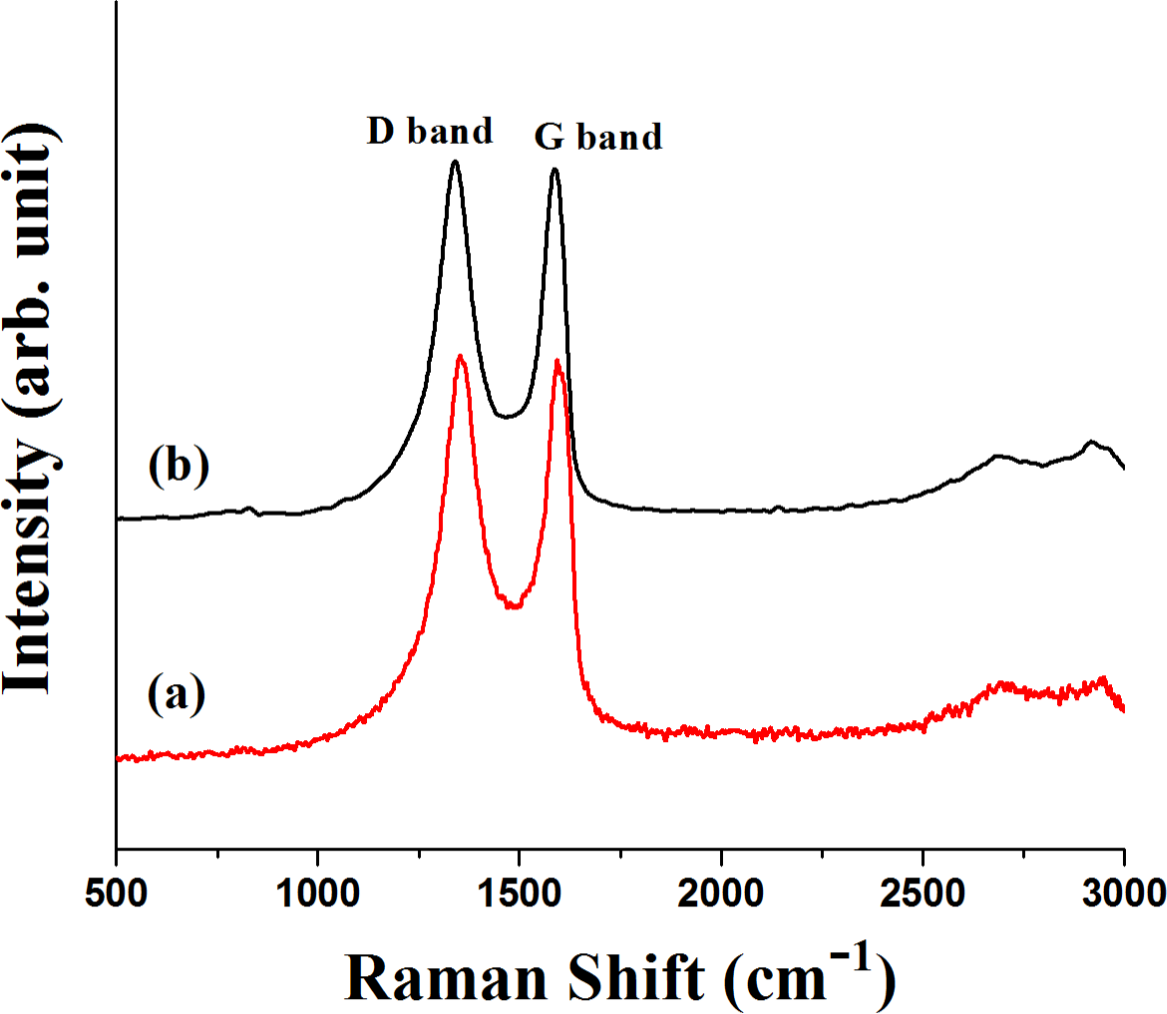
(a) Raman spectrum of fresh Ni/RGO-40 and (b) recovered catalyst after the first cycle of C–S coupling.

**Figure 3:**
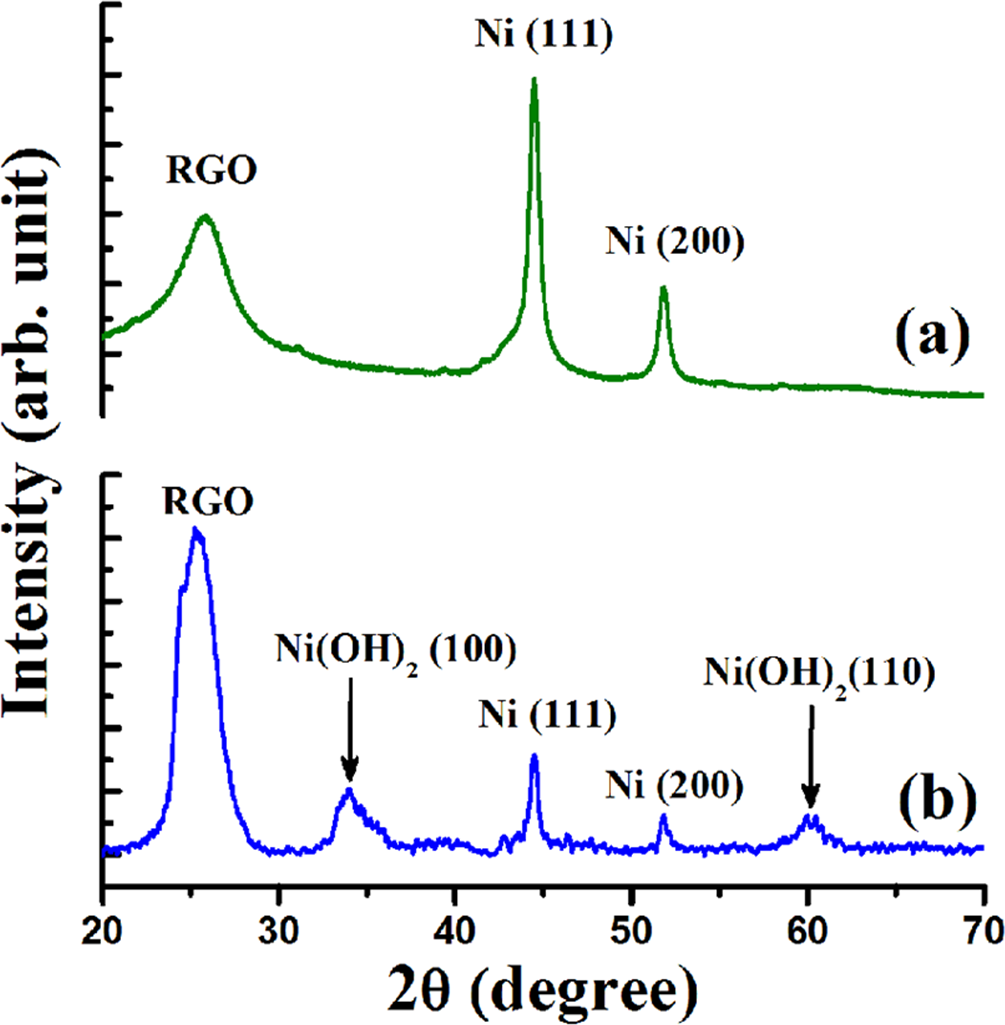
(a) XRD of fresh Ni/RGO-40 and (b) the recovered catalyst after the first cycle of C–S coupling.

**Figure 4:**
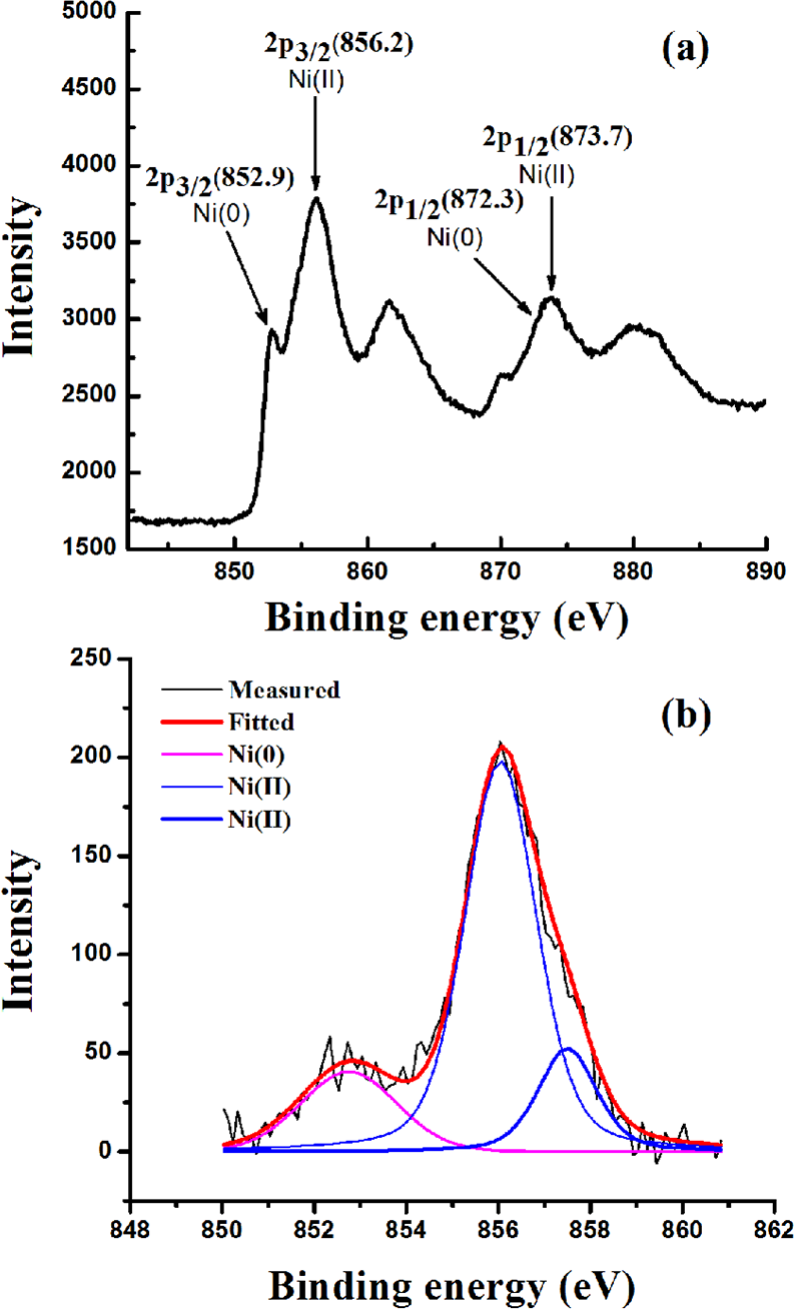
HRXPS of Ni in (a) Ni/RGO-40 catalyst recovered after the first cycle of the reaction. (b) Deconvoluted 2p_3/2_ peak of Ni from (a).

**Figure 5:**
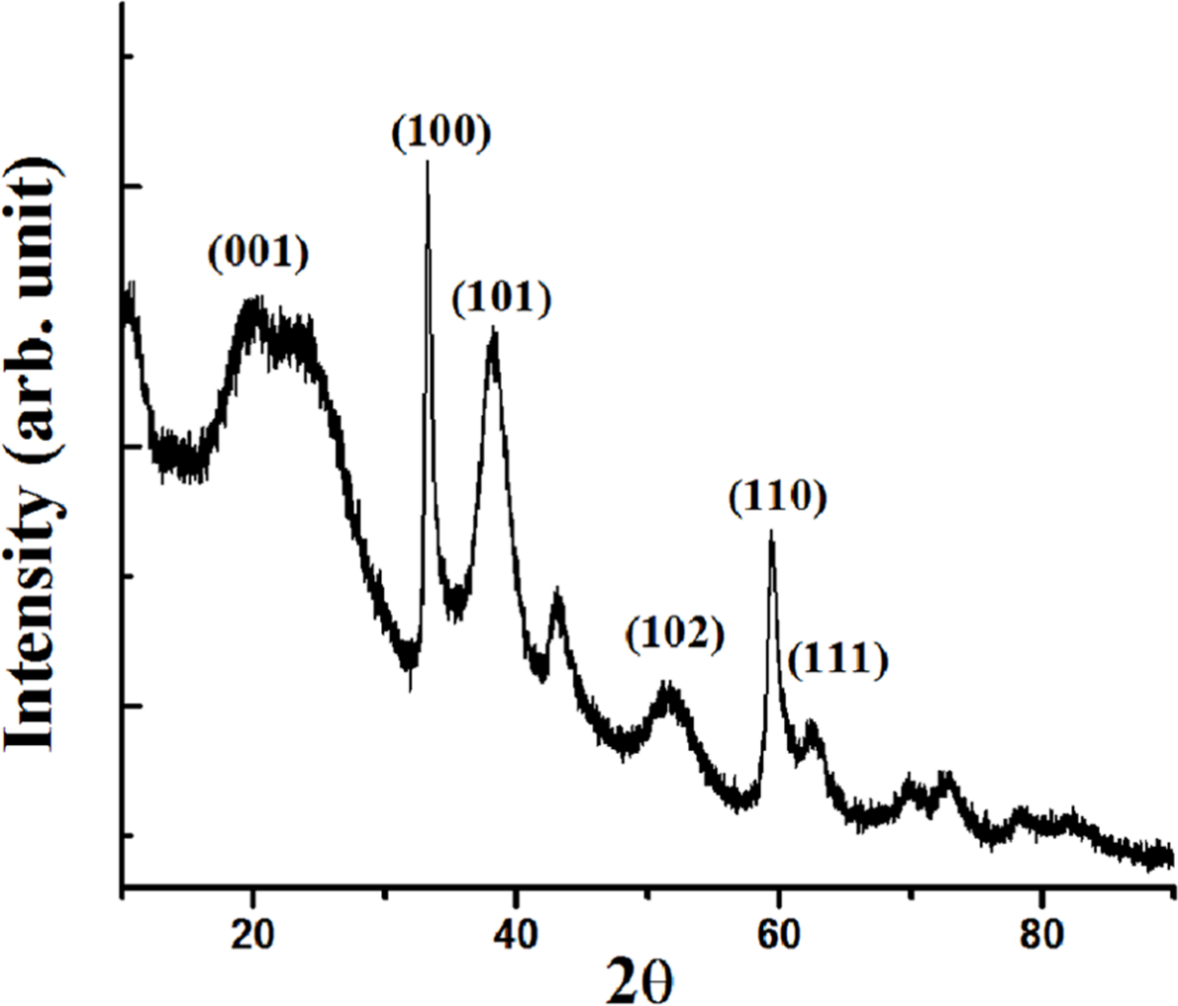
XRD of Ni(OH)_2_/RGO, prepared separately.

To establish the reaction mechanism, we characterized the recovered catalyst by Raman, XRD, XPS and TEM studies. The Raman and XRD results of fresh Ni/RGO-40 were reported in our previous publication [48]. However, for comparison, Raman and XRD of a fresh Ni/RGO-40 sample were again recorded and presented along with that of the recovered sample. The Raman spectrum of the Ni/RGO-40 composite (Figure 2a) exhibits a characteristic D band at 1344 cm^−1^ (A_1g_ vibrations of six-membered sp^2^ carbon rings) and the G band at 1580 cm^−1^ (first-order scattering of the E_2g_ mode of sp^2^ domains). After the first run, the Raman spectrum of the recovered Ni/RGO-40 catalyst (Figure 2b), however, did not reveal any peak related to the nickel oxide (NiO). The intensity ratio of the D over the G band was found to be 1.03, which is similar to that of Ni/RGO-40 before used in the C−S coupling reaction (Figure 2a). The powder XRD of Ni/RGO-40, recovered after the first cycle, was also recorded and is shown in Figure 3. The XRD pattern (Figure 3a) of the fresh sample shows peaks for Ni(111) and Ni(200) for Ni(0) being supported with RGO. The crystallite sizes of Ni(0) in the recovered catalyst were found to be 12 nm, calculated by using the X-ray line broadening method based on (111), (200) peaks in Figure 3b. The particle size clearly indicates that the planar surface of RGO effectively prevents the agglomeration of Ni NP during the catalysis. Apart from the characteristic peaks of Ni(0), two additional peaks were observed in the XRD of the recovered catalyst (Figure 3b). These two peaks indicate the presence of Ni(OH)_2_. It was further confirmed by performing X-ray photoelectron spectroscopy (XPS) of the recovered catalyst (Figure 4). The XPS of fresh catalyst shows only Ni(0) related peaks [48]. The HRXPS of Ni in the recovered Ni/RGO-40 (Figure 4a) clearly shows the peak at 852.9 eV corresponds to the 2p_3/2_ of the Ni(0) NPs [52]. Similarly the peak at 856.2 eV represents the 2p_3/2_ of Ni(OH)_2_ [52]. The deconvoluted spectrum (Figure 4b) shows that the recovered catalyst contain 17% Ni(0) and 83% Ni(II) species. It was interesting to observe that in the recycle runs, which proceeded with excellent conversion to the thioether, the catalytic system contains a significant amount of Ni(II) species embedded with RGO in addition to Ni(0) NPs. Therefore, assuming that Ni(II) species having embedded with RGO can also be used as the catalytic system, we prepared a new nanocomposite [Ni(OH)_2_/RGO (30 wt %)] [48], with average particle size of 13 nm (calculated on the basis of Figure 5) and examined its catalytic efficiency in the C–S cross-coupling reaction. We could achieve the C–S coupled thioether product in 82% yield only, which is lower than Ni/RGO-40 (see Supporting Information File 1, Table S1, a Table showing comparative catalytic efficiency and NP sizes).

**Figure 6:**
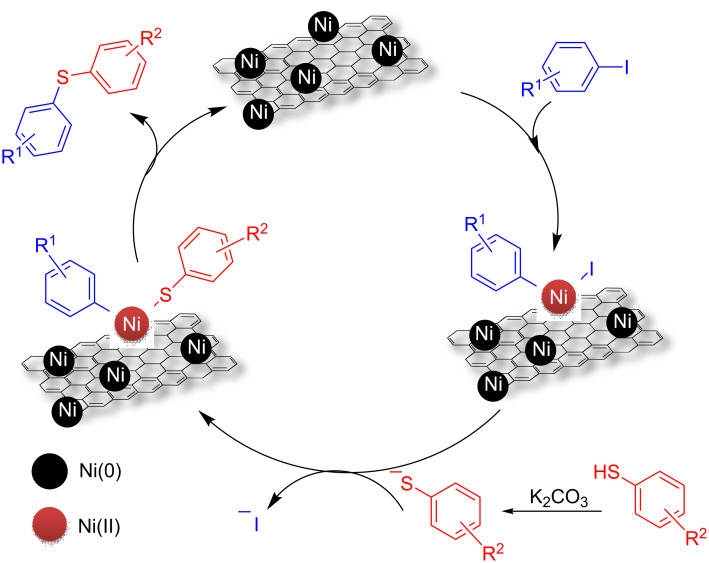
Proposed mechanism for the RGO-supported Ni-catalyzed C–S cross-coupling reaction.

**Figure 7:**
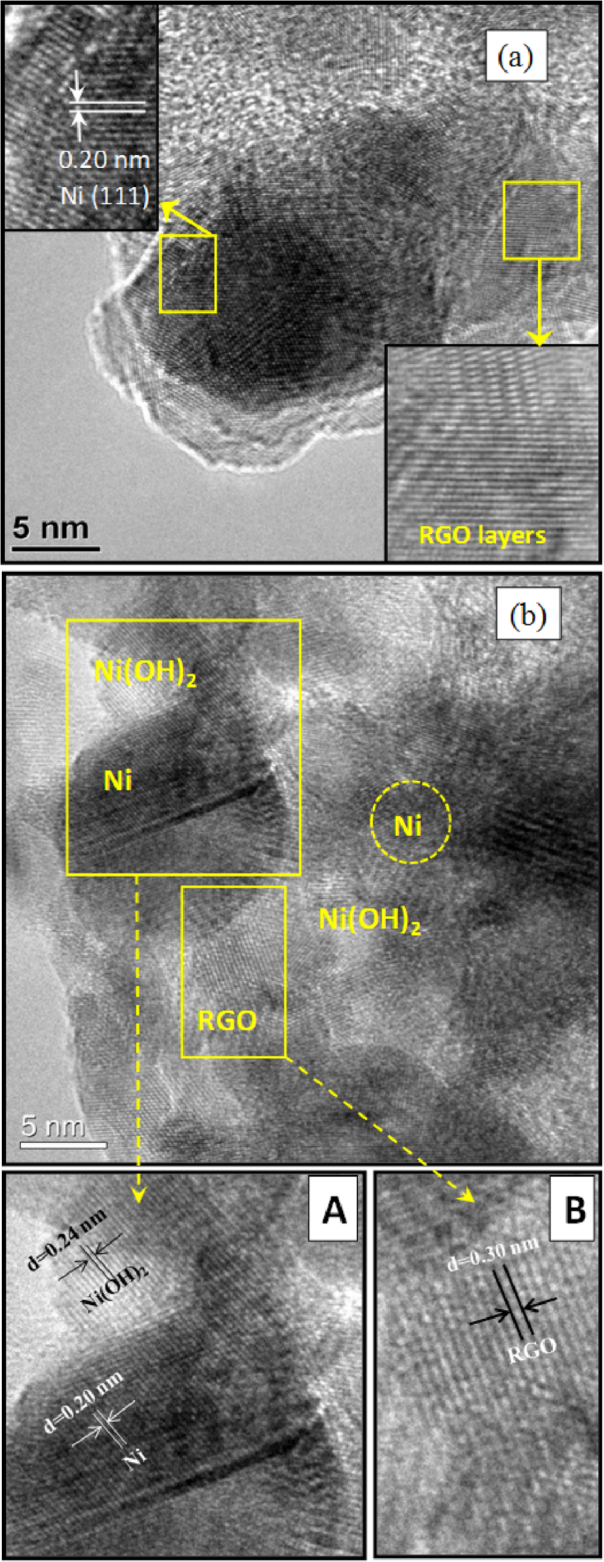
TEM image of (a) Ni/RGO-40 before usage as a catalyst (for comparison, reprinted with permission from [48], copyright 2014 American Chemical Society) and (b) recovered catalyst after the first cycle of reaction showing the different domains (marked by yellow enclosures). The enlarged views of the marked rectangular areas in (b) are shown in A (Ni species) and B (RGO).

Mechanistically, the transition-metal-catalyzed C–S cross-coupling reaction, using mainly Pd, Cu or Ni species, is believed to proceed through three major steps; viz. the initial oxidative addition, then substitution by thiolate anion and finally the reductive elimination. A more detailed study describing Pd- or Cu-catalyzed C–S coupling reactions as compared to Ni-catalyzed reaction has been published [7,53–54]. However, as proposed in previous investigations on the Ni-catalyzed C–S coupling reaction [15,22–23], the catalytic cycle here is likely to occur in analogy. Examination of the recovered catalyst by HRXPS suggests that there is significant quantity of Ni(II) hydroxide and interestingly that is also equally active in catalyzing the C–S coupling reaction. Initial oxidative addition to an Ar–I bond gives Ar–Ni^II^–I followed by Ar–Ni^II^–SPh, and then the reductive elimination could result in the formation of Ar–SPh (Figure 6). The resulting Ni(II) species could be converted mostly to Ni(OH)_2_ NPs in the presence of water during washing and subsequent recovery, as examined from the powder XRD and HRXPS of the recovered catalyst after the first run (Figure 3b and Figure 4b). In support of our proposed reaction mechanism, we analyzed the TEM images of the fresh Ni/RGO-40 catalyst and the recovered one after the first cycle of catalysis. In Figure 7a, we show the TEM image of fresh Ni/RGO-40 (figure reproduced from our earlier publication, ref [48]) for comparison and that of the recovered Ni/RGO-40 is shown in Figure 7b. Figure 7b clearly shows the presence of metallic Ni NPs and Ni(OH)_2_ on the RGO surface. One can observe the co-existence of crystalline fringes of Ni<111> and Ni(OH)_2_<100> (inset A; Figure 7b) along with the interlayer spacing of RGO (inset B; Figure 7b). The particle size of Ni NPs observed from TEM image was almost similar in the before and after catalysis sample, i.e., around 11 nm, indicating the particle agglomeration did not occur during the catalysis. Again, the crystallite size of the Ni(OH)_2_ was found to be around 4 nm, which is corroborated from the X-ray line broadening method on the <100> peak of Ni(OH)_2_ in Figure 3b. It is likely that Ni(OH)_2_ could form during the recovery of the catalyst. In the recycle run, the major catalytic constituent Ni(OH)_2_ is presumably reduced by thiolate anions to Ni(I) species, which then undergoes oxidative addition to the aryl halide forming a Ni(III) species, as proposed previously [19].

## Conclusion

In conclusion, we have shown that a RGO-supported Ni(0) nanocomposite (Ni/RGO-40) with average size (≈11–12 nm) Ni NPs display high catalytic efficiency for C–S cross-coupling reactions and are applicable to a diverse range of coupling partners. The catalytic performance is primarily dependent on the NP sizes of the Ni species. The electron-rich planar surface of RGO helps in stabilizing the NPs and prevents agglomeration making them recyclable with almost equal efficiency. However, post-catalytic investigations of the heterogeneous catalyst reveal that the Ni NPs are considerably converted to Ni(OH)_2_, (average particle size 4 nm) and remain closely associated with the Ni NPs on the electron-rich RGO surface and exhibit similar catalytic efficiency.

## Experimental

Chemicals were used as received. For column chromatography, silica (60–120 mm, SRL, India) was used. For thin-layer chromatography (TLC), plates (Merck) coated with silica gel 60, F_254_ were used.

The progress of the reaction was monitored by HPLC (Agilent Technologies, 1260 Infinity), Column: ZORBAX Rx–SIL (4.6 × 150 mm, 5 mm), eluent: *n*-hexane (flow rate 2 mL min^−1^). ^1^H and ^13^C NMR spectra were taken in CDCl_3_ using a Bruker Avance AV-300 spectrometer operating at 300 MHz and 75 MHz, respectively. Chemical shifts are reported relative to tetramethylsilane (TMS) served as the internal standard (δ = 0 ppm). ^13^C NMR spectra were recorded with complete proton decoupling and chemical shifts are reported in ppm with the solvent resonance as the internal standard (CDCl_3_: δ = 77.00 ppm). Raman spectra were obtained using Renishaw InVia Reflex micro Raman spectrometer with excitation of argon ion (514 nm) lasers. The laser power was kept sufficiently low to avoid heating of the samples and spectra were collected with a resolution of 1 cm^−1^. X-ray diffraction (XRD) studies of the powder samples were performed with Rigaku Smartlab X-ray diffractometer operating at 9 kW (200 mA; 45 kV) using Cu Kα radiation. X-ray photoelectron spectroscopic (XPS) measurements were done on a PHI 5000 Versaprobe II XPS system with Al Kα source and a charge neutralizer at room temperature, maintaining a base pressure about 6 × 10^−10^ mbar and energy resolution of 0.6 eV.

### General procedure for C–S cross-coupling using Ni/RGO-40

A mixture of aryl halide (1 mmol), thiol (1.2 mmol), potassium carbonate (1.2 mmol), Ni/RGO-40 catalyst (22 mg; Ni content is 8.8 mg, 0.15 mmol, 15 mol %) in DMF (3 mL) were taken in a 15 mL sealed tube, flashed and filled with N_2_ gas and quickly screw-capped. The reaction mixture was then heated to 100 °C with a gentle magnetic stirring for hours. After completion of the reaction, the mixture was allowed to cool, diluted with ethyl acetate (3 mL), stirred gently and then allowed to stand for 15 min. The supernatant liquid was carefully pipetted out into another flask and this process was repeated three more times. The organic part was washed with water, dried over anhydrous Na_2_SO_4_, concentrated to afford a residue, which was purified by column chromatography over a short column of silica gel and eluting with light petroleum to obtain pure sulfanes. All unsymmetrical sulfanes were characterized by ^1^H and ^13^C NMR and compared with the reported data (see Supporting Information File 1, pages S3–S6).

In order to recover the catalyst, the insoluble materials obtained after washing with ethyl acetate were thoroughly washed with water (3 × 3 mL) followed by acetone (3 × 3 mL) and then dried under vacuum to obtain a free-flowing black powder. This material was used for the next catalytic cycle.

### Gram-scale procedure for C–S cross-coupling using Ni/RGO-40

A mixture of 4-iodoanisole (1 g, 4.27 mmol), thiophenol (0.563 g, 5.12 mmol), potassium carbonate (0.706 g, 5.12 mmol), Ni/RGO-40 catalyst (94.0 mg; Ni content is 37.60 mg, 0.64 mmol) in DMF (4 mL) were taken in a 15 mL sealed tube, flashed and filled with N_2_ gas and quickly screw-capped. The reaction mixture was then heated to 100 °C with a gentle magnetic stirring for 3 hours. After cooling the reaction mixture was diluted with ethyl acetate (4 mL), stirred gently and then allowed to stand for 15 min. The supernatant liquid was carefully pipetted out into another flask and this process was repeated three more times. The organic part was washed with water, dried over anhydrous Na_2_SO_4_, and concentrated to afford a residue, which was purified by column chromatography over a short column of silica gel and eluting with light petroleum to obtain 0.85 g (92%) of pure (4-methoxyphenyl)(phenyl)sulfane as a colourless liquid.

## Supporting Information

File 1 Powder XRD patterns of Ni/RGO-20 and Ni/RGO-40; Table showing comparative catalytic performance and effect of NP sizes; ^1^H and ^13^C NMR spectral data for compounds **3a**–**p**.

File 2 ^1^H and ^13^C NMR spectra (scanned) for compounds **3a**–**p**.
