# Supplementary material for: Ni nanoparticles on RGO as reusable heterogeneous catalyst: effect of Ni particle size and intermediate composite structures in C–S cross-coupling reaction
Source: Beilstein J Org Chem. 2017 Aug 28;13:1796–806. doi: 10.3762/bjoc.13.174 (PMC5588615; doi:10.3762/bjoc.13.174)
Supplement: File 1 — Powder XRD patterns of Ni/RGO-20 and Ni/RGO-40; Table showing comparative catalytic performance and effect of NP sizes; 1H and 13C NMR spectral data for compounds 3a–p. [file Beilstein_J_Org_Chem-13-1796-s001.pdf]

Supporting Information

for

Ni nanoparticles on RGO as reusable heterogeneous catalyst: effect of Ni particle size and intermediate composite structures in C–S cross-coupling reaction

Debasish Sengupta[‡1], Koushik Bhowmik[‡2], Goutam De[*2] and Basudeb Basu[*1]

Address: [1]Department of Chemistry, University of North Bengal, Darjeeling 734013, India. Fax: +91 353 2699001; Tel: +91 353 2776381 and [2]Nano-Structured Materials Division, CSIR–Central Glass & Ceramic Research Institute, 196, Raja S. C. Mullick Road, Jadavpur, Kolkata 700032, India. Fax: +91 33 24730957; Tel: +91 33 23223403.

Email: Goutam De[*] - gde@cgcri.res.in; Basudeb Basu[*] - basu_nbu@hotmail.com,

*Corresponding author

[‡]These authors have contributed equally

Powder XRD patterns of Ni/RGO-20 and Ni/RGO-40; Table showing comparative catalytic performance and effect of NP sizes; ^{1}H and ^{13}C NMR spectral data for compounds 3a–p

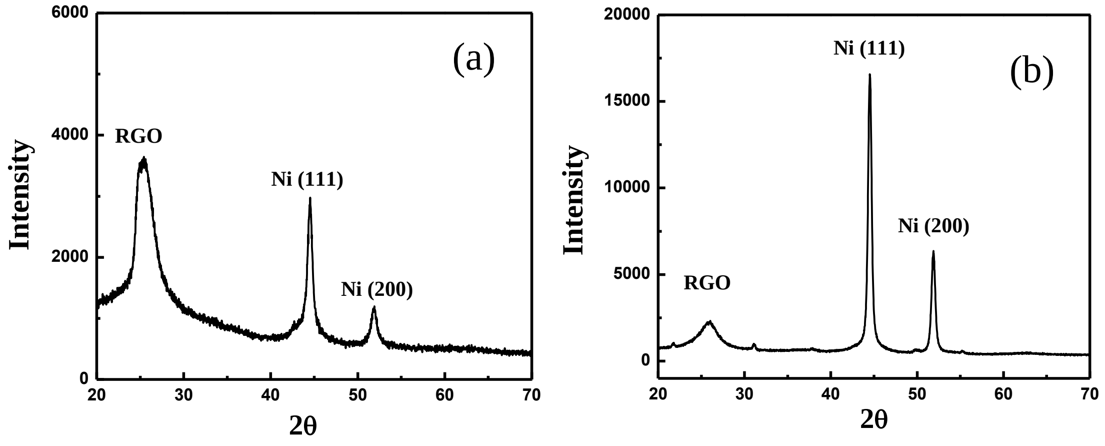

Figure S1: P−XRD patterns of (a) Ni/RGO−20 and (b) Ni/RGO−60.

Table S1: Comparative Table showing catalytic performance and NP sizes.

Comparison of various heterogeneous nickel catalysts in C–S cross-coupling reaction

Entry	Ni-Catalyst	Average particle size (nm)	Amount of Ni (mmol)	Temp (°C)	Time (h)	Yield (%)	TOF (h^{-1})	Ref.
1	Ni/RGO–40	11	0.15	100	2	93[a]	3.10	–
2	After 1st cycle Ni/Ni$(OH)_2$/RGO	12 & 4	0.15	100	2	93[a]	3.10	–
3	Ni$(OH)_2$/RGO (30 wt %)	13	0.15	100	2	82[a]	2.73	–
4	NiO–ZrO_2	7	0.078	80	24	89[b]	0.47	§

[a]Isolated yield of (4–Methoxyphenyl)(phenyl)sulfane.

[b]Isolated yield of (4–Chlorophenyl)(4–methoxyphenyl)sulfane.

§ Pal, N.; Bhaumik, A. *Dalton Trans*. **2012**, *41*, 9161–9169.

NMR spectral data for compounds listed in Table 2

(4-Methoxyphenyl)(phenyl)sulfane (3a)[1]

Colourless liquid

^{1}H NMR ($CDCl_3$, 300 MHz): δ/ppm 3.82 (s, 3H, OC**H**$_3$), 6.90 (dd, J = 2.1 and 6.6 Hz, 2H, Ar**H**), 7.14–7.26 (m, 5H, Ar**H**), 7.42 (dd, J = 2.1, 6.9 Hz, 2H, Ar**H**); ^{13}C NMR ($CDCl_3$, 75 MHz): δ/ppm 55.3, 114.9, 124.1, 125.7, 128.1, 128.9, 135.3, 138.6, 159.7.

(3-Methoxyphenyl)(phenyl)sulfane (3b)[2]

Colourless liquid

^{1}H NMR ($CDCl_3$, 300 MHz): δ/ppm 3.71 (s, 3H, OC**H**$_3$), 6.75 (dd, J = 2.7 and 8.4 Hz, 1H, Ar**H**), 6.85-6.91 (m, 2H, Ar**H**), 7.14-7.37 (m, 6H, Ar**H**); ^{13}C NMR ($CDCl_3$, 75 MHz): δ/ppm 55.1, 112.6, 115.8, 122.8, 127.1, 129.1, 129.8, 131.3, 135.2, 137.1, 159.9.

(2-Methoxyphenyl)(*p*–tolyl)sulfane (3c)[1]

Colourless liquid

^{1}H NMR ($CDCl_3$, 300 MHz): δ/ppm 2.35 (s, 3H, C**H**$_3$), 3.88 (s, 3H, OC**H**$_3$), 6.80–6.88 (m, 2H, Ar**H**), 6.94 (dd, J = 1.5 and 7.8 Hz, 1H, Ar**H**), 7.13–7.20 (m, 3H, Ar**H**), 7.31 (d, J = 8.1 Hz, 2H, Ar**H**); ^{13}C NMR ($CDCl_3$, 75 MHz): δ/ppm 21.1, 55.8, 110.6, 121.2, 125.7, 127.4, 129.7, 129.8, 130.1, 132.9, 137.7, 156.5.

(4-Fluorophenyl)(4-methoxyphenyl)sulfane (3d)[3]

Colourless liquid

^{1}H NMR ($CDCl_3$, 300 MHz): δ/ppm 3.80 (s, 3H, OC**H**$_3$), 6.86-6.98 (m, 4H, Ar**H**), 7.17-7.22 (m, 2H, Ar**H**), 7.34-7.37 (m, 2H, Ar**H**); ^{13}C NMR ($CDCl_3$, 75 MHz): δ/ppm 55.3, 115.0, 116.0 (d, $^{2}J_{C-F}$ = 21.8 Hz), 125.2, 131.0 (d, $^{2}J_{C-F}$ = 7.9 Hz) , 133.1, 134.5, 159.6, 161.6 (d, $^{2}J_{C-F}$ = 243.4 Hz).

(4-Methoxyphenyl)(*p*-tolyl)sulfane (3e)[4]

Colourless liquid

^{1}H NMR ($CDCl_3$, 300 MHz): δ/ppm 2.29 (s, 3H, C**H**$_3$), 3.79 (s, 3H, OC**H**$_3$), 6.86 (d, *J* = 9.0 Hz, 2H, Ar**H**), 7.05 (d, *J* = 8.1 Hz, 2H, Ar**H**), 7.13 (d, *J* = 8.1 Hz, 2H, Ar**H**), 7.35 (d, *J* = 9.0 Hz, 2H, Ar**H**); ^{13}C NMR ($CDCl_3$, 75 MHz): δ/ppm 20.9, 55.3, 114.8, 125.6, 129.3, 129.7, 134.3, 136.1, 159.4.

(4-Methoxyphenyl)(2,5-dimethylphenyl)sulfane (3f)[5]

Colourless liquid

^{1}H NMR ($CDCl_3$, 300 MHz): δ/ppm 2.20 (s, 3H, C**H**$_3$), 2.32 (s, 3H, C**H**$_3$), 3.80 (s, 3H, OC**H**$_3$), 6.85–6.92 (m, 4H, Ar**H**), 7.06 (d, *J* = 7.5 Hz, 1H, Ar**H**), 7.29 (dd, *J* = 2.1 and 6.6 Hz, 2H, Ar**H**); ^{13}C NMR ($CDCl_3$, 75 MHz): δ/ppm 19.8, 20.9, 55.3, 114.9, 124.9, 127.2, 130.1, 130.2, 133.9, 134.3, 136.0, 136.1, 159.2.

1-(4-(Phenylthio)phenyl)ethanone (3g)[2]

White solid, mp 62-63 (Lit. mp 63-64 ^{o}C)

^{1}H NMR ($CDCl_3$, 300 MHz): δ/ppm 2.52 (s, 3H, COC**H**$_3$), 7.20 (d, *J* = 8.7 Hz, 2H, Ar**H**), 7.36-7.49 (m, 5H, Ar**H**), 7.78-7.81 (m, 2H, Ar**H**); ^{13}C NMR ($CDCl_3$, 75 MHz): δ/ppm 26.3, 127.3, 128.6, 128.7, 129.5, 131.9, 133.7, 134.3, 144.7, 196.9.

(3-Nitrophenyl)(phenyl)sulfane (3h)[6]

Yellowish liquid

^{1}H NMR ($CDCl_3$, 300 MHz): δ/ppm 7.41-7.53 (m, 7H, Ar**H**), 8.00-8.05 (m, 2H, Ar**H**); ^{13}C NMR ($CDCl_3$, 75 MHz): δ/ppm 120.9, 123.1, 128.9, 129.6, 129.8, 132.1, 133.4, 134.2, 140.6, 148.7.

(4-Chlorophenyl)(3-nitrophenyl)sulfane (3i)[6]

Yellow solid, mp 72-73 (Lit. mp 70-71 ^{o}C)

^{1}H NMR ($CDCl_3$, 300 MHz): δ/ppm 7.36-7.51 (m, 6H, Ar**H**), 8.03-8.06 (m, 2H, Ar**H**); ^{13}C NMR ($CDCl_3$, 75 MHz): δ/ppm 121.3, 123.4, 129.8, 130.0, 130.8, 134.4, 134.5, 135.1, 139.7, 148.6.

(3-Bromophenyl)(*p*-tolyl)sulfane (3j)[7]

Colourless liquid

^{1}H NMR ($CDCl_3$, 300 MHz): δ/ppm 2.36 (s, 3H, C**H**$_3$), 7.10–7.18 (m, 4H, Ar**H**), 7.24–7.28 (m, 1H, Ar**H**), 7.32–7.35 (m, 3H, Ar**H**); ^{13}C NMR ($CDCl_3$, 75 MHz): δ/ppm 21.2, 122.9, 127.2, 129.0, 129.4, 130.2, 130.3, 131.1, 133.2, 138.5, 140.3.

(3-Chlorophenyl)(*p*-tolyl)sulfane (3k)[8]

Colourless liquid

^{1}H NMR ($CDCl_3$, 300 MHz): δ/ppm 2.36 (s, 3H, C**H**$_3$), 7.07–7.19 (m, 6H, Ar**H**), 7.34 (dd, *J* = 1.8 and 6.3 Hz, 2H, Ar**H**); ^{13}C NMR ($CDCl_3$, 75 MHz): δ/ppm 21.2, 126.1, 126.7, 128.3, 129.4, 129.9, 130.3, 133.3, 134.8, 138.6, 140.0.

(4-Methoxy-3-methylphenyl)(phenyl)sulfane (3l)

Colourless liquid

^{1}H NMR ($CDCl_3$, 300 MHz): δ/ppm 2.22 (s, 3H, C**H**$_3$), 3.87 (s, 3H, OC**H**$_3$), 6.85-7.35 (m, 8H, Ar**H**); ^{13}C NMR ($CDCl_3$, 75 MHz): δ/ppm 16.1, 55.4, 110.8, 123.6, 125.6, 128.1, 128.9, 132.9, 136.3, 138.9, 158.1.

1,2-Bis(*p*-tolylthio)benzene (3m)[9]

White solid, mp 76-77 ^{o}C (Lit mp. 75.5 ^{o}C)

^{1}H NMR ($CDCl_3$, 300 MHz): δ/ppm 2.34 (s, 6H, C**H**$_3$), 7.04-7.05 (m, 4H, Ar**H**), 7.13-7.17 (m, 4H, Ar**H**), 7.28-7.31 (m, 4H, Ar**H**); ^{13}C NMR ($CDCl_3$, 75 MHz): δ/ppm 21.1, 127.0, 130.1, 130.5, 132.5, 137.5, 137.8.

1,3-Bis(*p*-tolylthio)benzene (3n)[1]

White solid, mp 84–85 °C

^{1}H NMR ($CDCl_3$, 300 MHz): *δ*/ppm 2.34 (s, 6H, C**H**$_3$), 6.98–7.01 (m, 2H, Ar**H**), 7.06–7.13 (m, 6H, Ar**H**), 7.27 (dd, *J* = 1.5 and 6.3 Hz, 4H, Ar**H**); ^{13}C NMR ($CDCl_3$, 75 MHz): *δ*/ppm 21.1, 126.6, 129.0, 129.3, 130.1, 132.9, 138.0, 138.7.

(4-Methoxyphenyl)(pentyl)sulfane (3o)[10]

Colourless liquid

^{1}H NMR ($CDCl_3$, 300 MHz): *δ*/ppm 0.88 (t, *J* = 7.2 Hz, 3H, C**H**$_3$), 1.26–1.40 (m, 4H, C**H**$_2$–C**H**$_2$), 1.56–1.61 (m, 2H, C**H**$_2$), 2.81 (m, 2H, S–C**H**$_2$), 3.79 (s, 3H, OC**H**$_3$), 6.83 (dd, *J* = 2.1 and 6.6 Hz, 2H, Ar**H**), 7.33 (dd, *J* = 2.1 and 6.6 Hz, 2H, Ar**H**); ^{13}C NMR ($CDCl_3$, 75 MHz): *δ*/ppm 13.9, 22.2, 29.0, 30.8, 35.7, 55.3, 114.4, 126.9, 132.9, 158.6.

(3-Methoxyphenyl)(heptyl)sulfane (3p)

Colourless liquid

^{1}H NMR ($CDCl_3$, 300 MHz): *δ*/ppm 0.85–0.90 (m, 3H, C**H**$_3$), 1.26–1.68 (m, 10H, –(C**H**$_2$)$_5$–, 2.91 (t, *J* = 7.2 Hz, 2H, S–C**H**$_2$), 3.79 (s, 3H, OC**H**$_3$), 6.696 (ddd, *J* = 0.9, 2.4 and 8.1 Hz, 1H, Ar**H**), 6.85–6.91(m, 2H, Ar**H**), 7.16–7.25 (m, 1H, Ar**H**); ^{13}C NMR ($CDCl_3$, 75 MHz): *δ*/ppm 14.0, 22.6, 28.8, 29.1, 31.3, 31.7, 33.3, 55.2, 111.2, 114.0, 120.8, 129.6, 138.5, 159.8.

References

1. Basu, B.; Mandal, B.; Das, S.; Kundu, S. *Tetrahedron Lett*. **2009**, *50*, 5523–5528. doi: 10.1016/j.tetlet.2009.07.076

2. Park, N.; Park, K.; Jang, M.; Lee, S. *J. Org. Chem*. **2011**, *76*, 4371–4378. doi: 10.1021/jo2007253

3. Lin, Y.; Cai, M.; Fang, Z.; Zhao, H. *Tetrahedron* **2016**, *72*, 3335–3343. doi: 10.1016/j.tet.2016.04.063

4. Jammi, S.; Sakthivel, S.; Rout, L.; Mukherjee, T.; Mandal, S.; Mitra, R.; Saha, P.; Punniyamurthy, T. *J. Org. Chem*. **2009**, *74*, 1971−1976. doi: 10.1021/jo8024253

5. Fernandez–Rodriguez, M. A.; Shen, Q.; Hartwig, J. F. *Chem. Eur. J*. **2006**, *12*, 7782−7796. doi: 10.1021/jo0003347

6. Wu, W. -Y.; Wang, J. -C.; Tsai, F. -Y. *Green. Chem*. **2009**, *11*, 326−329. doi: 10.1039/B820790A

7. Campbell, J. R. *J. Org. Chem*. **1962**, *27*, 2207−2209. doi: 10.1021/jo01053a504

8. Sengupta, D.; B, Basu. *Org. Med. Chem. Lett.* **2014**, 4:17. Doi: 10.1186/s13588-014-0017-7

9. Nakayama, J.; Tajiri, T.; Hoshino, M. *Bull. Chem, Soc. Jpn*. **1986**, *59*, 2907−2908. doi: 10.1246/bcsj.59.2907

10. Suter, C. M.; Hansen, H. L. *J. Am. Chem. Soc*. **1932**, *54*, 4100−4104. doi: 10.1021/ja01349a039
