# Supplementary material for: Ni nanoparticles on RGO as reusable heterogeneous catalyst: effect of Ni particle size and intermediate composite structures in C–S cross-coupling reaction
Source: Beilstein J Org Chem. 2017 Aug 28;13:1796–806. doi: 10.3762/bjoc.13.174 (PMC5588615; doi:10.3762/bjoc.13.174)

Supporting Information

for

Ni nanoparticles on RGO as reusable heterogeneous catalyst: effect of Ni particle size and intermediate composite structures in C–S cross-coupling reaction

Debasish Sengupta[‡1], Koushik Bhowmik[‡2], Goutam De[*2] and Basudeb Basu[*1]

Address: [1]Department of Chemistry, University of North Bengal, Darjeeling 734013, India. Fax: +91 353 2699001; Tel: +91 353 2776381 and [2]Nano-Structured Materials Division, CSIR–Central Glass & Ceramic Research Institute, 196, Raja S. C. Mullick Road, Jadavpur, Kolkata 700032, India. Fax: +91 33 24730957; Tel: +91 33 23223403.

Email: Goutam De[*] - gde@cgcri.res.in; Basudeb Basu[*] - basu_nbu@hotmail.com,

*Corresponding author

‡These authors have contributed equally

^{1}H and ^{13}C NMR spectra (scanned) for compounds 3a–p

Table 2; entry 1

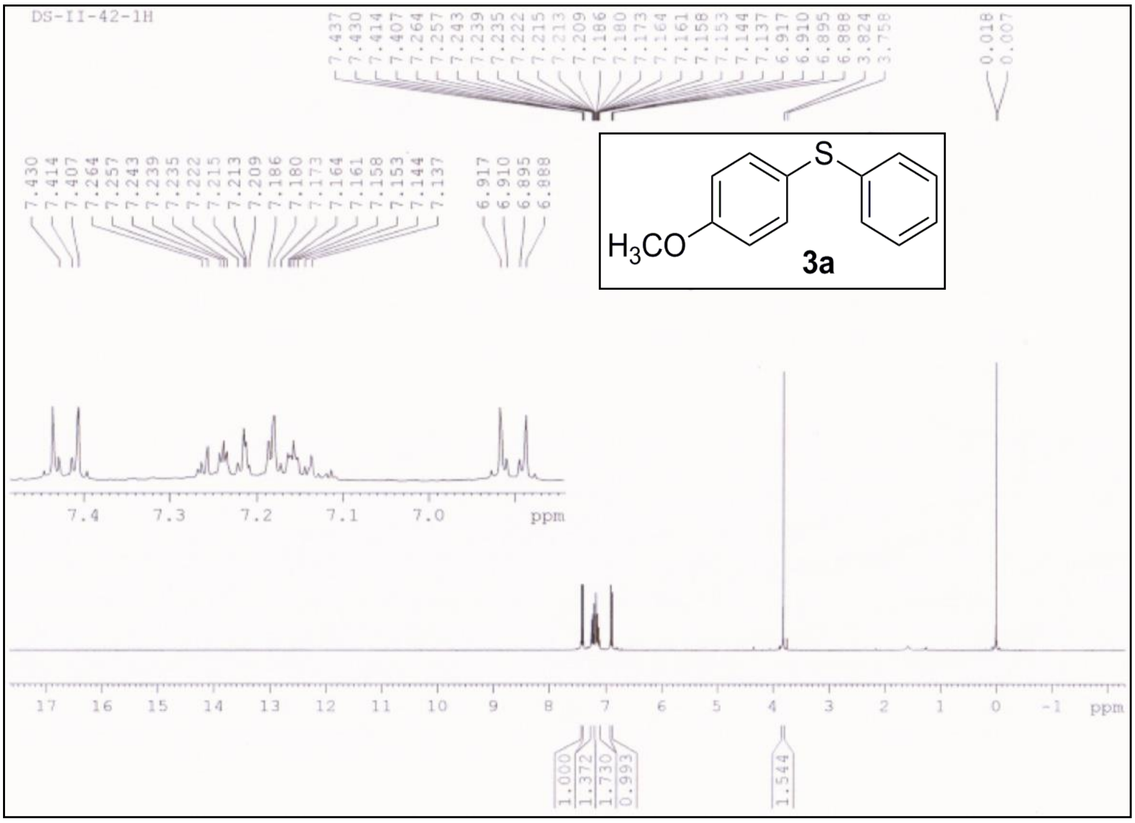

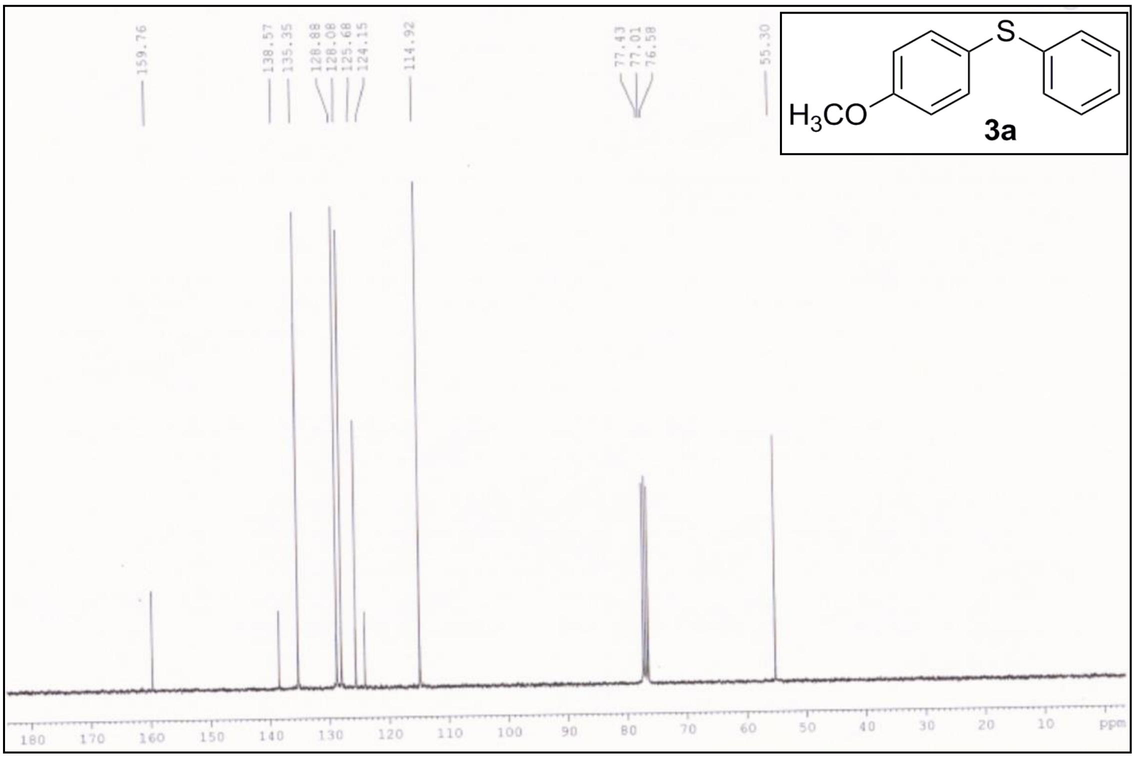

Table 2; entry 2

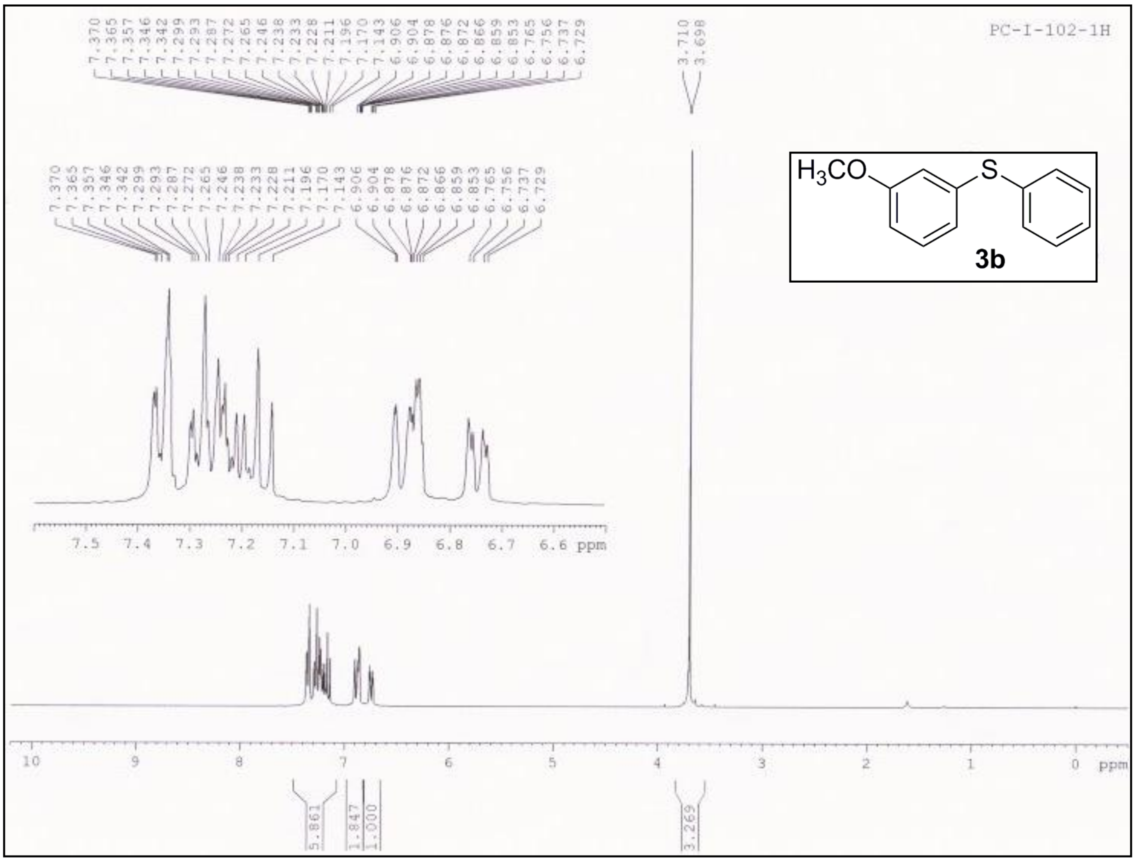

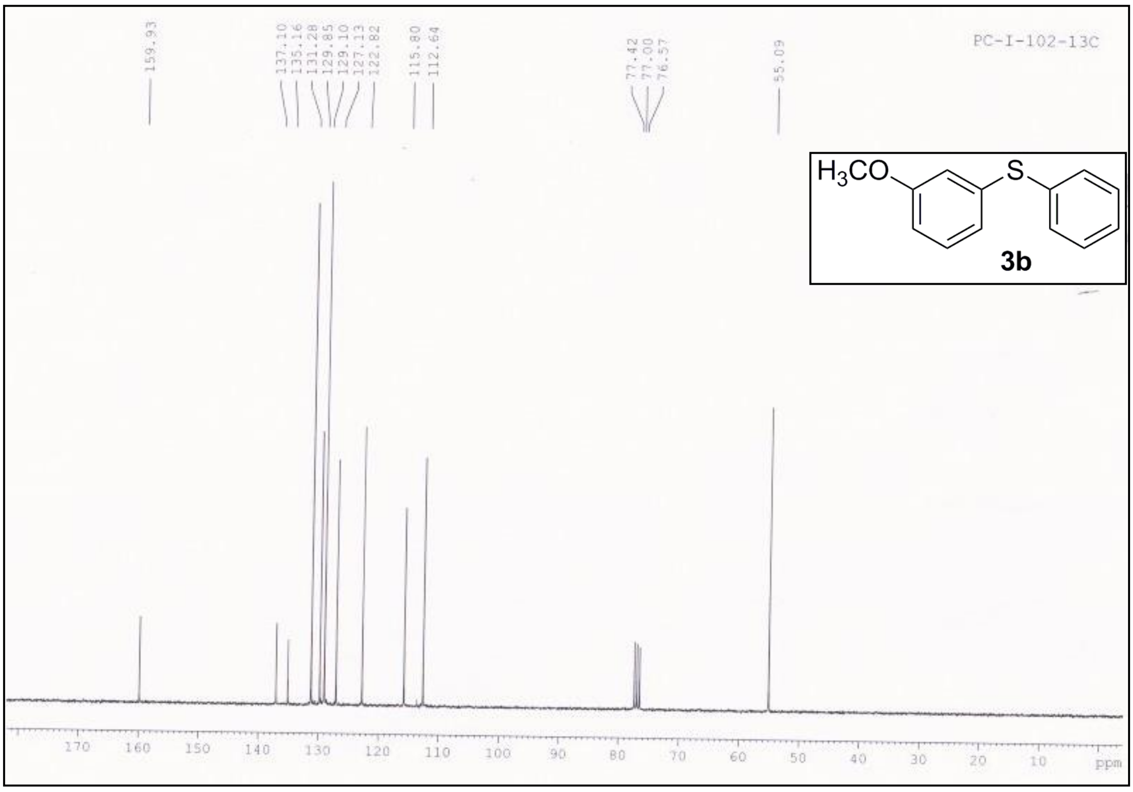

Table 2; entry 3

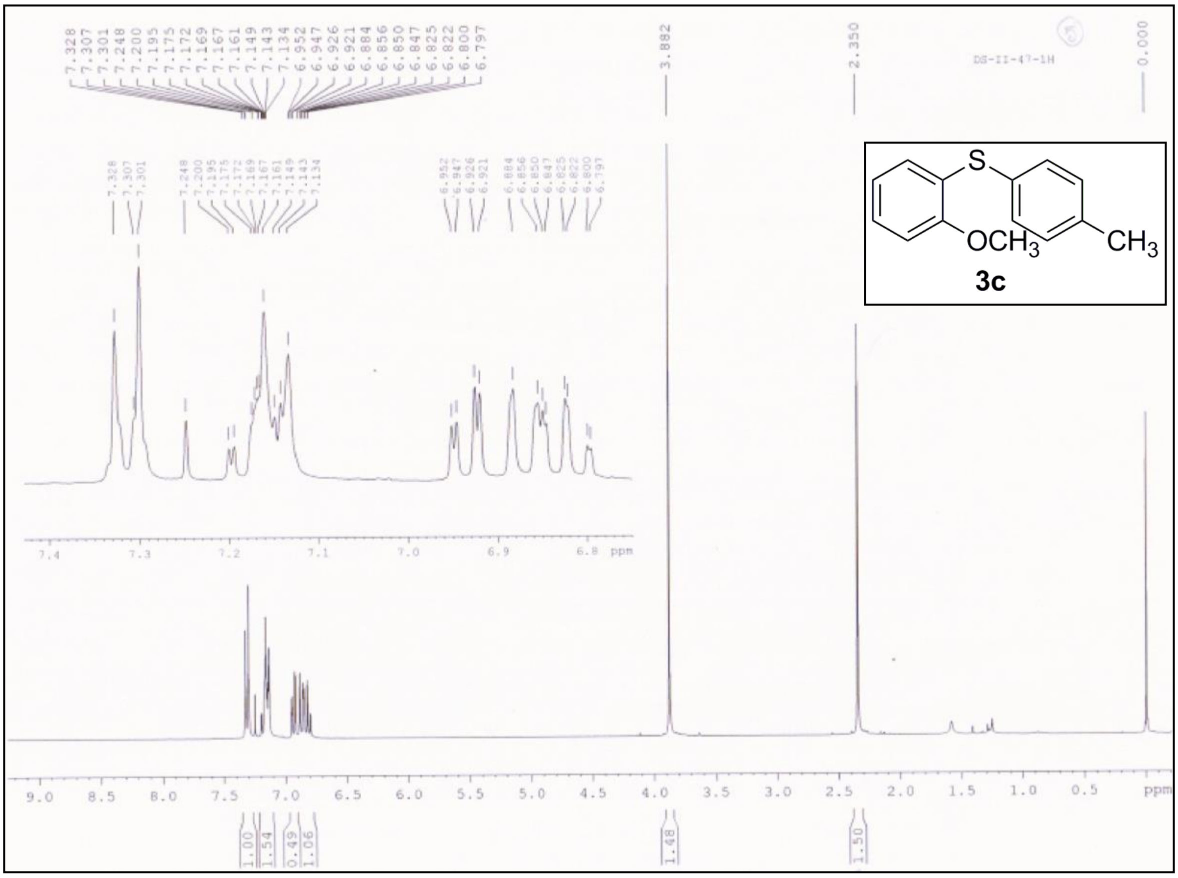

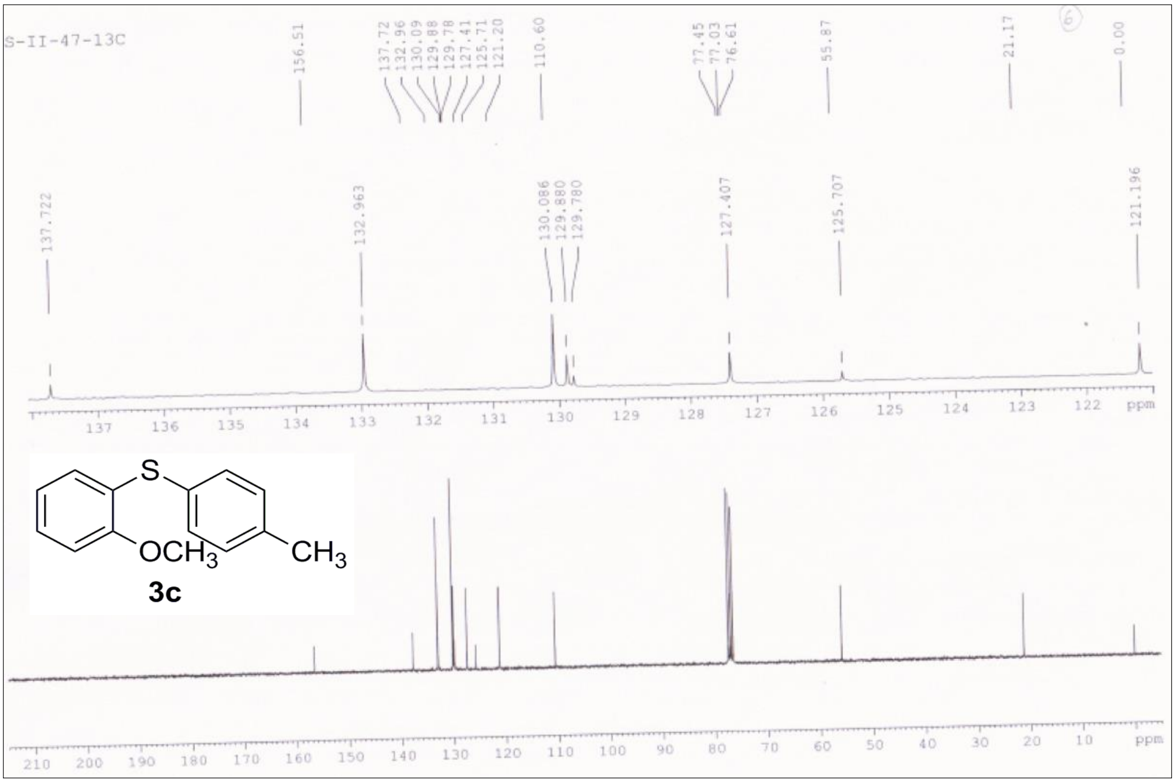

Table 2; entry 4

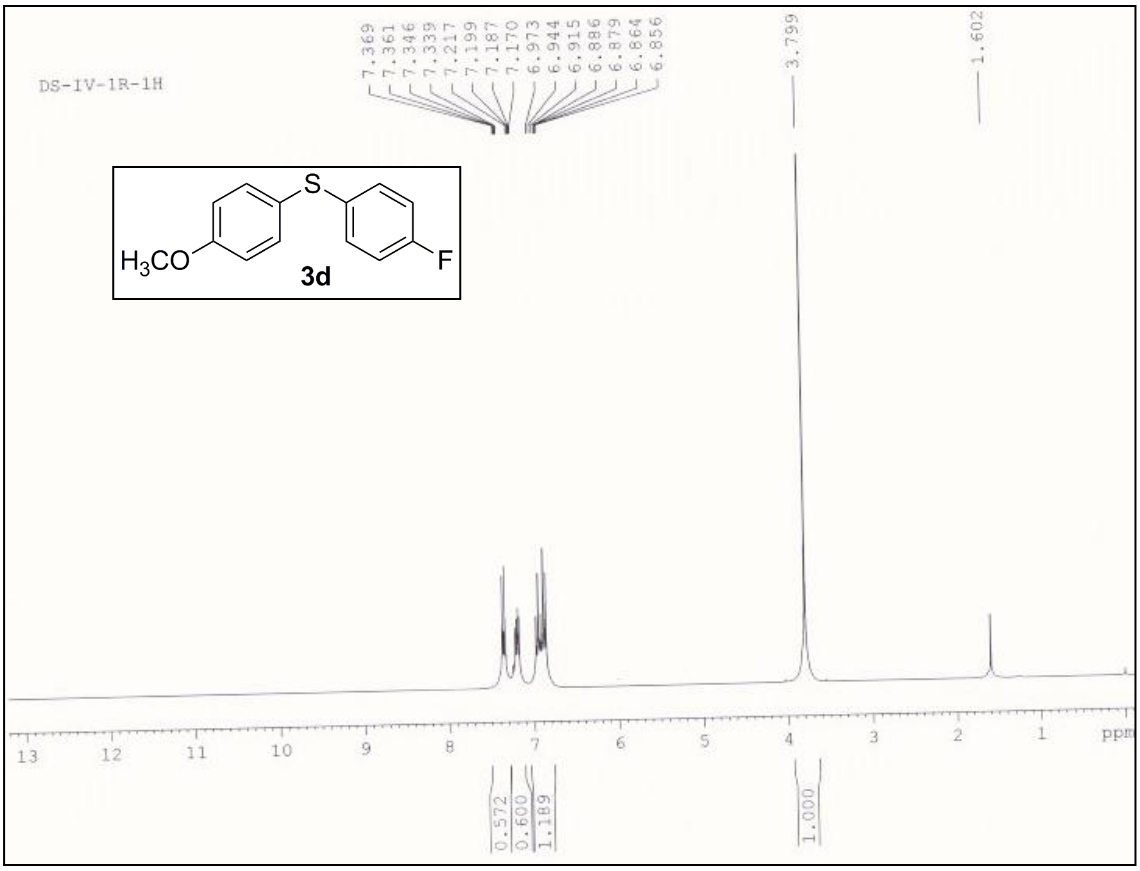

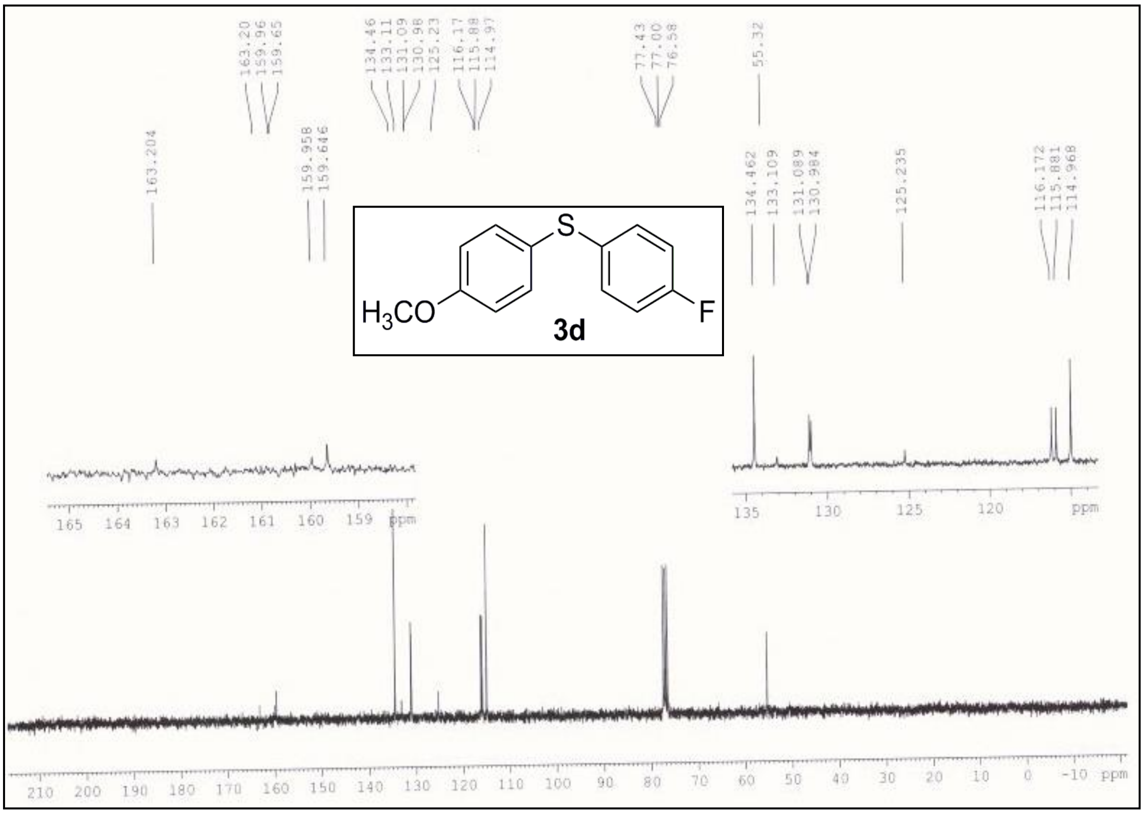

Table 2; entry 5

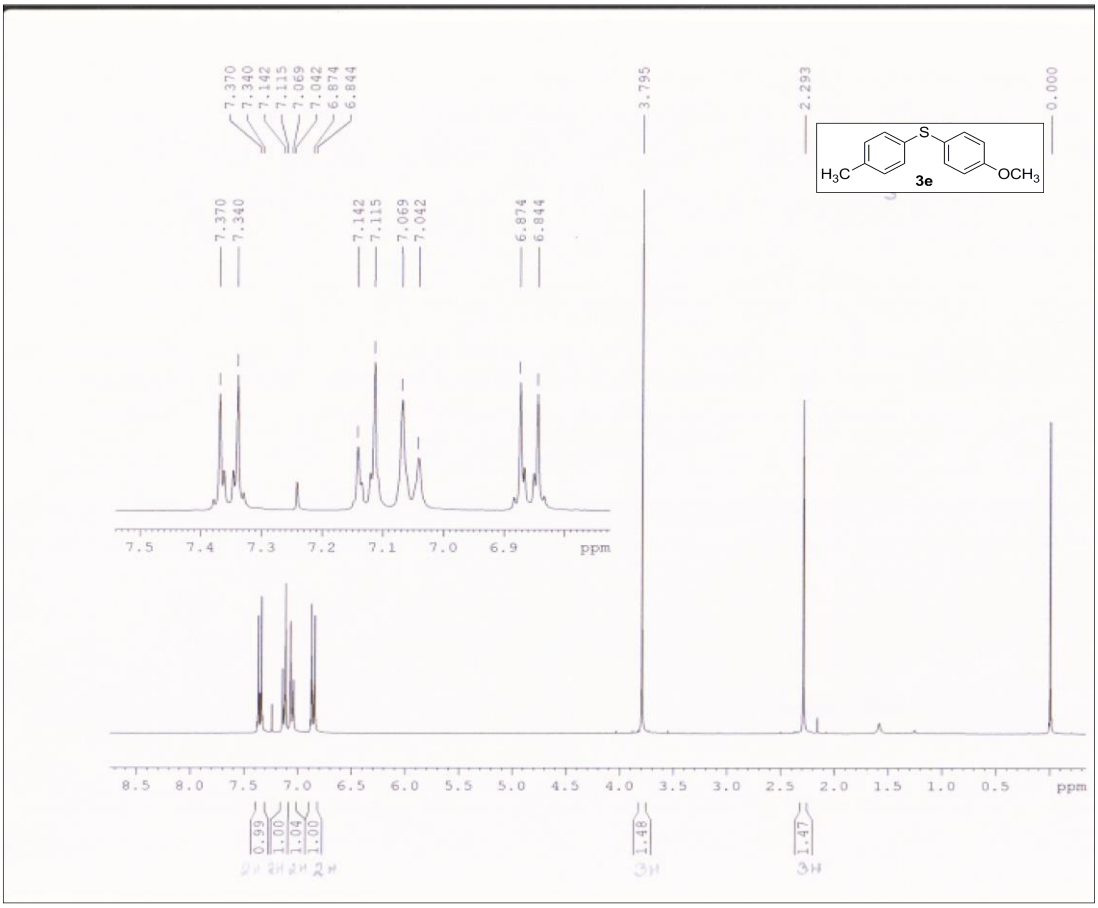

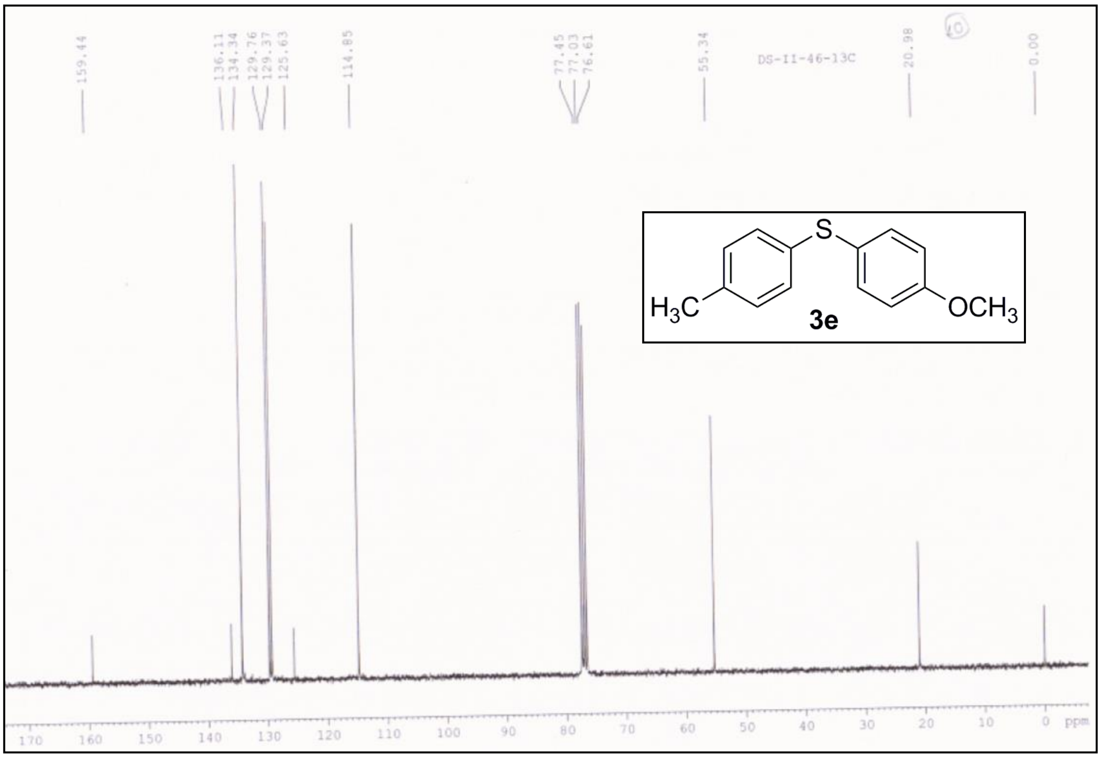

Table 2; entry 6

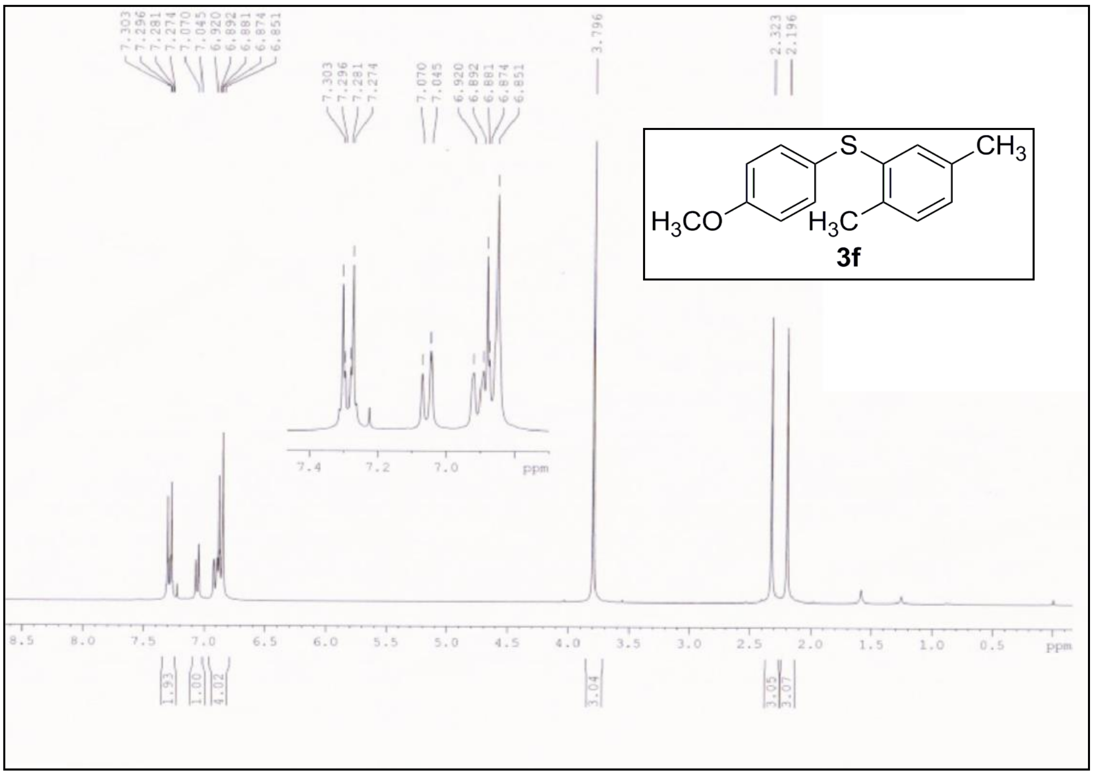

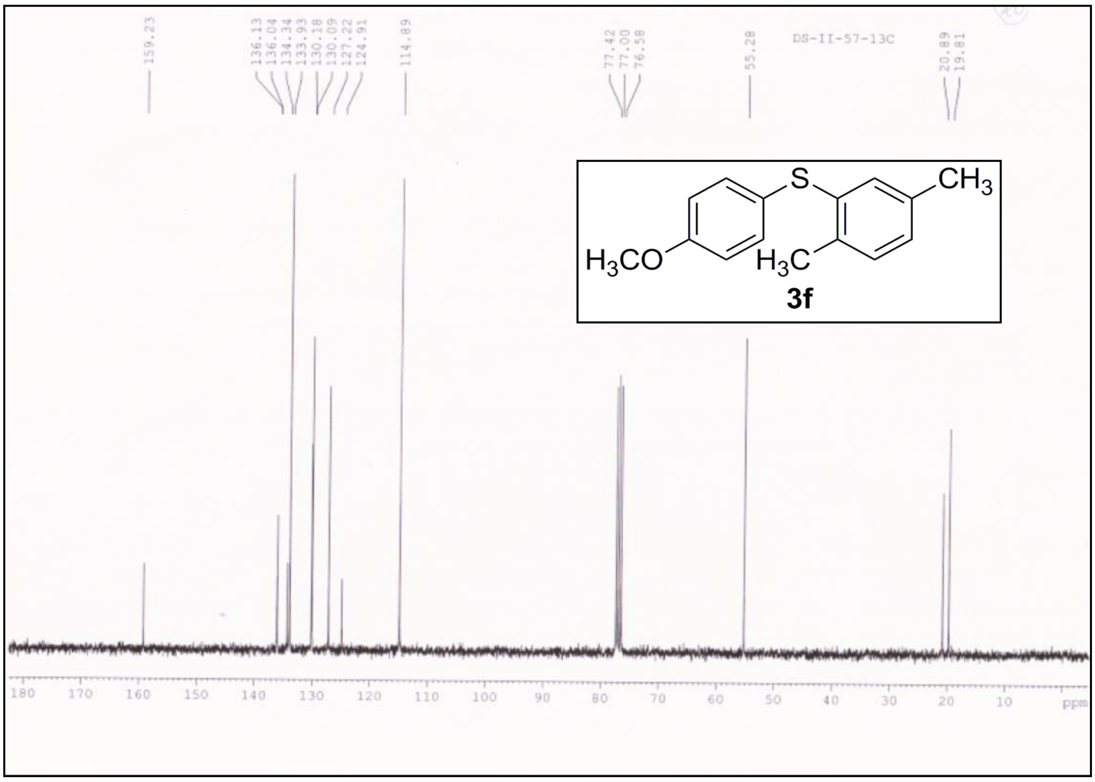

Table 2; entry 7

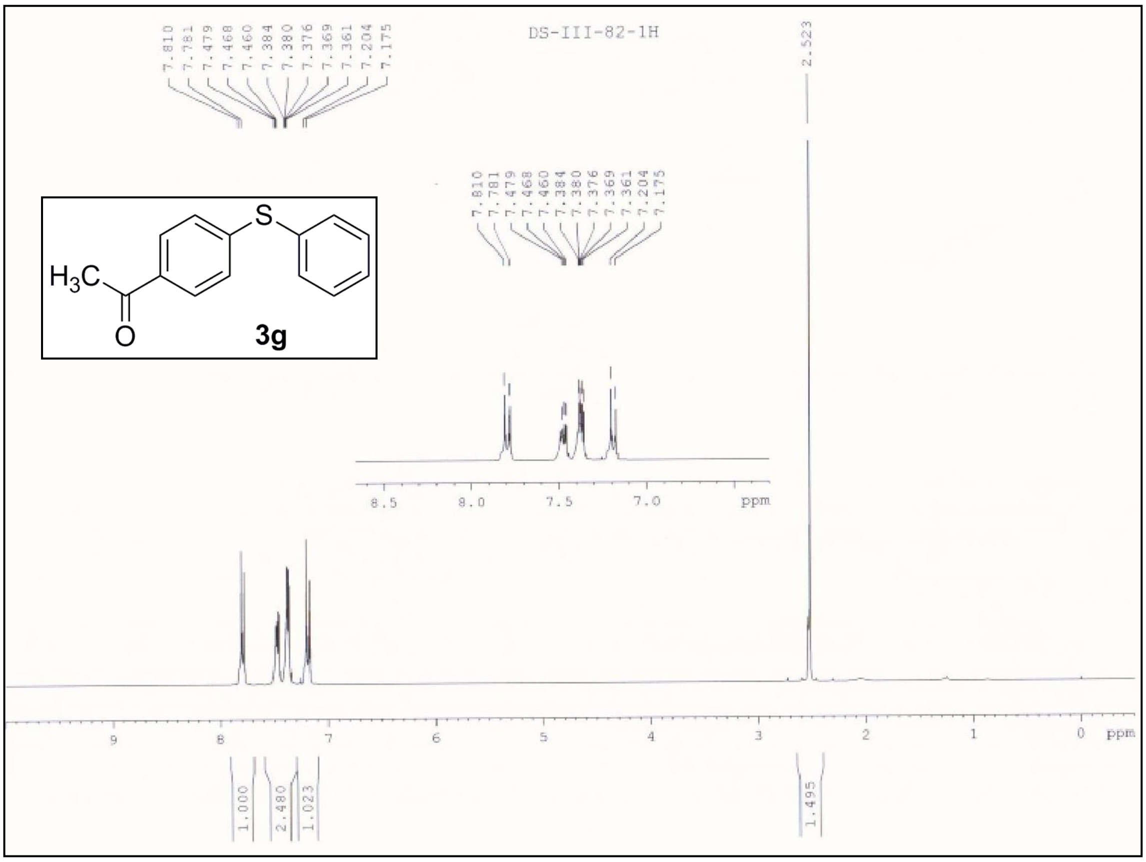

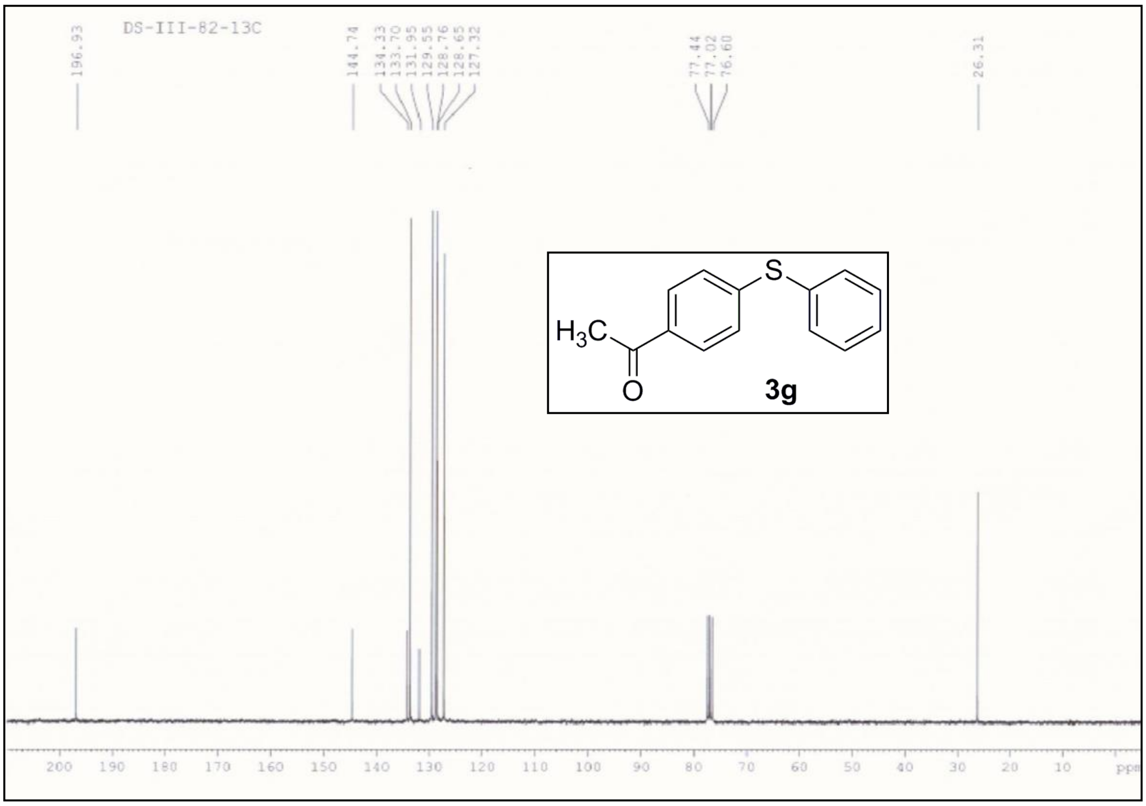

Table 2; entry 8

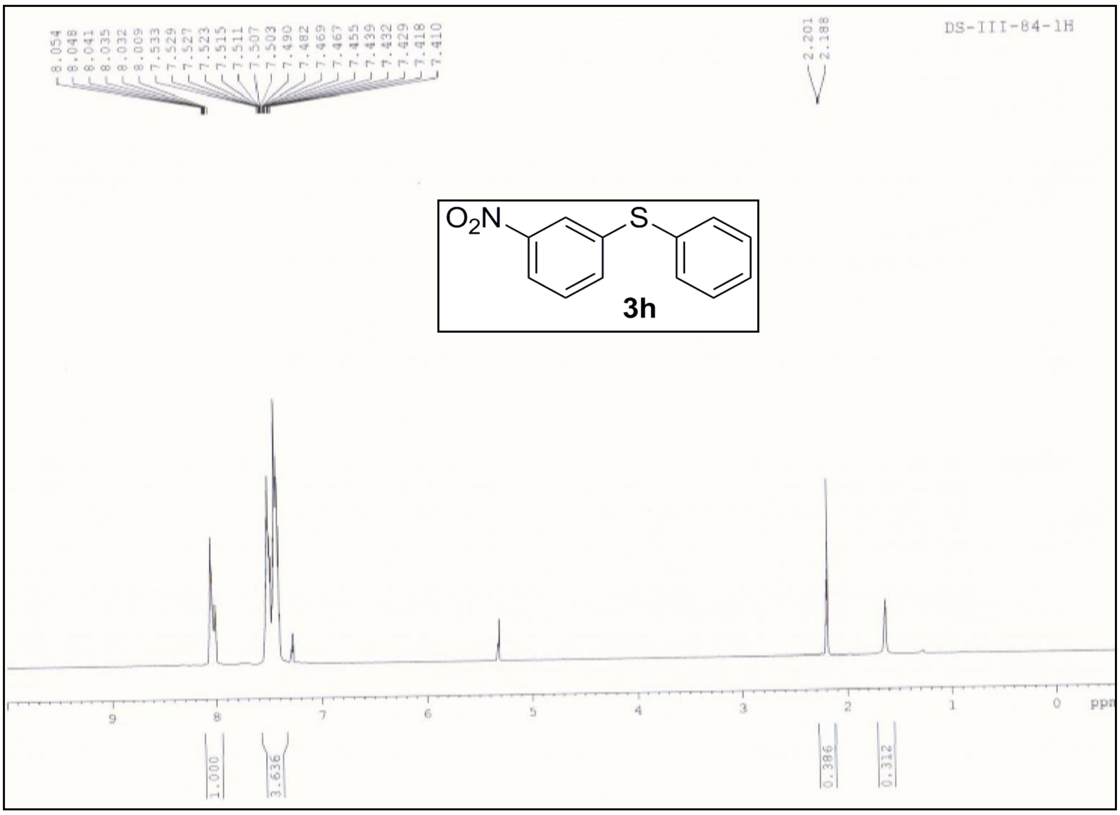

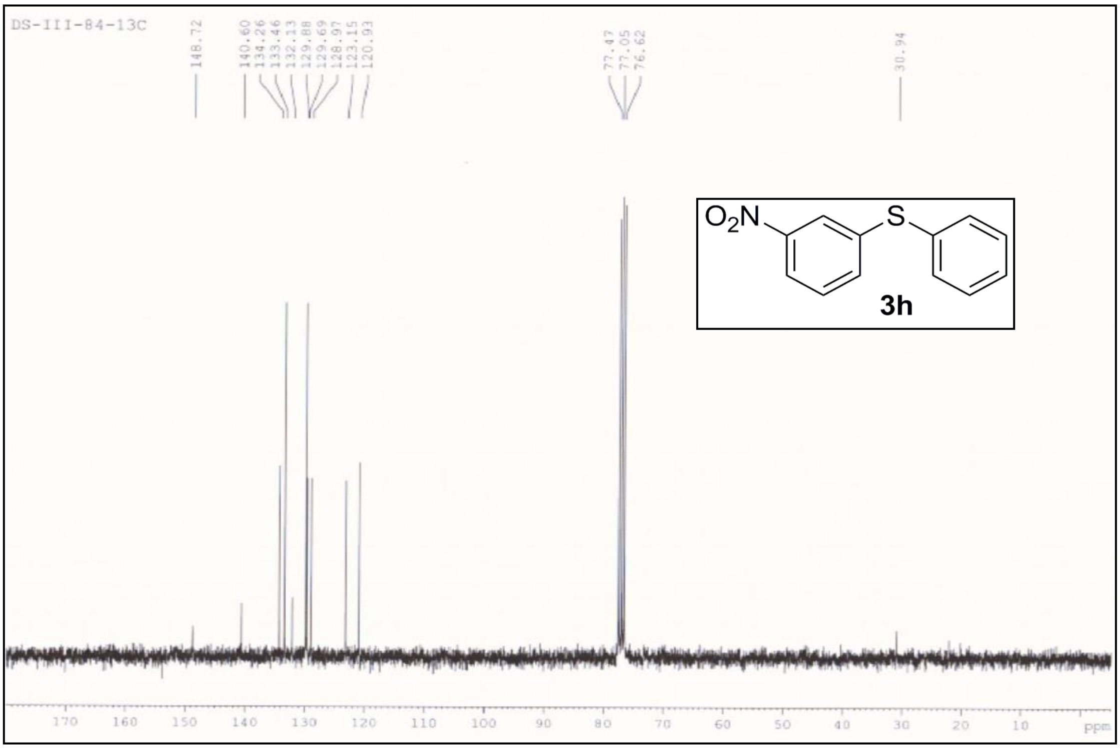

Table 2; entry 9

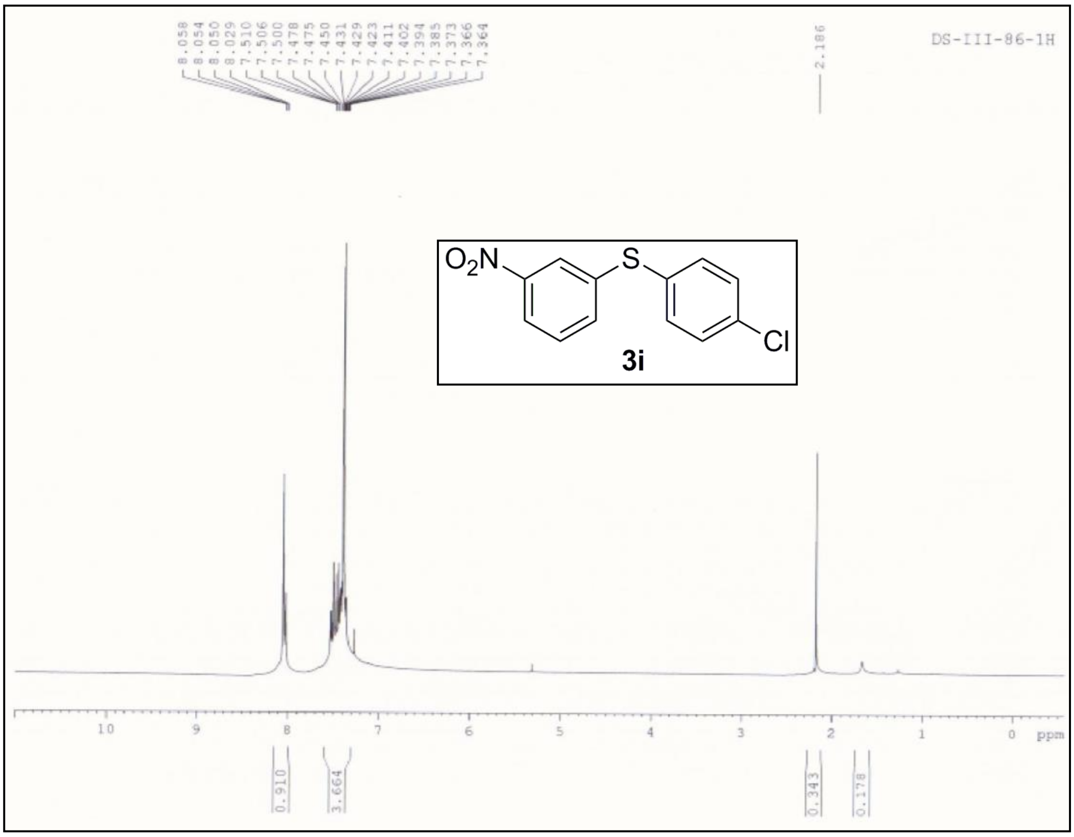

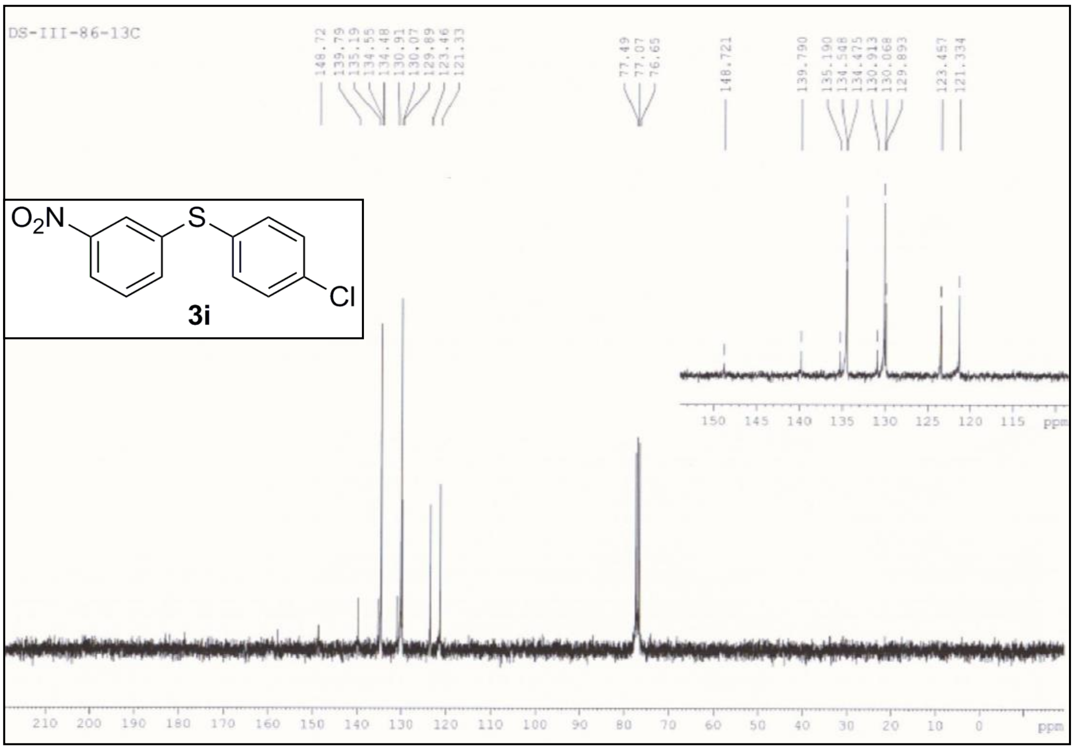

Table 2; entry 10

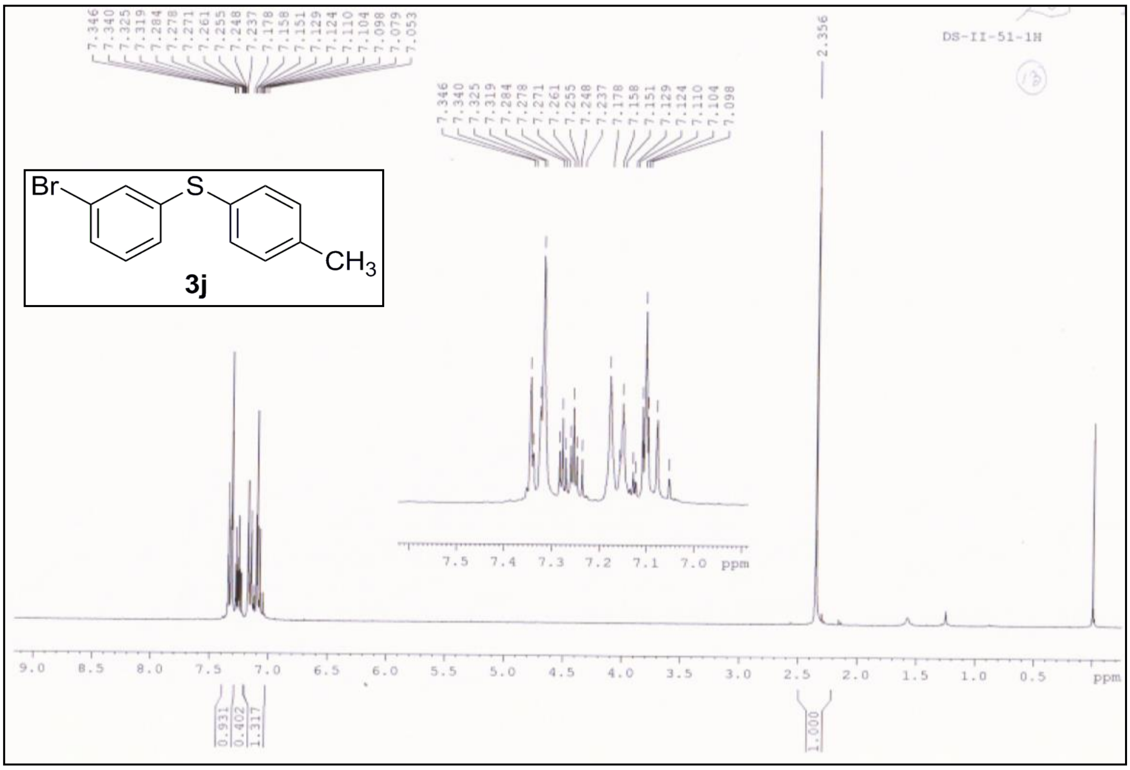

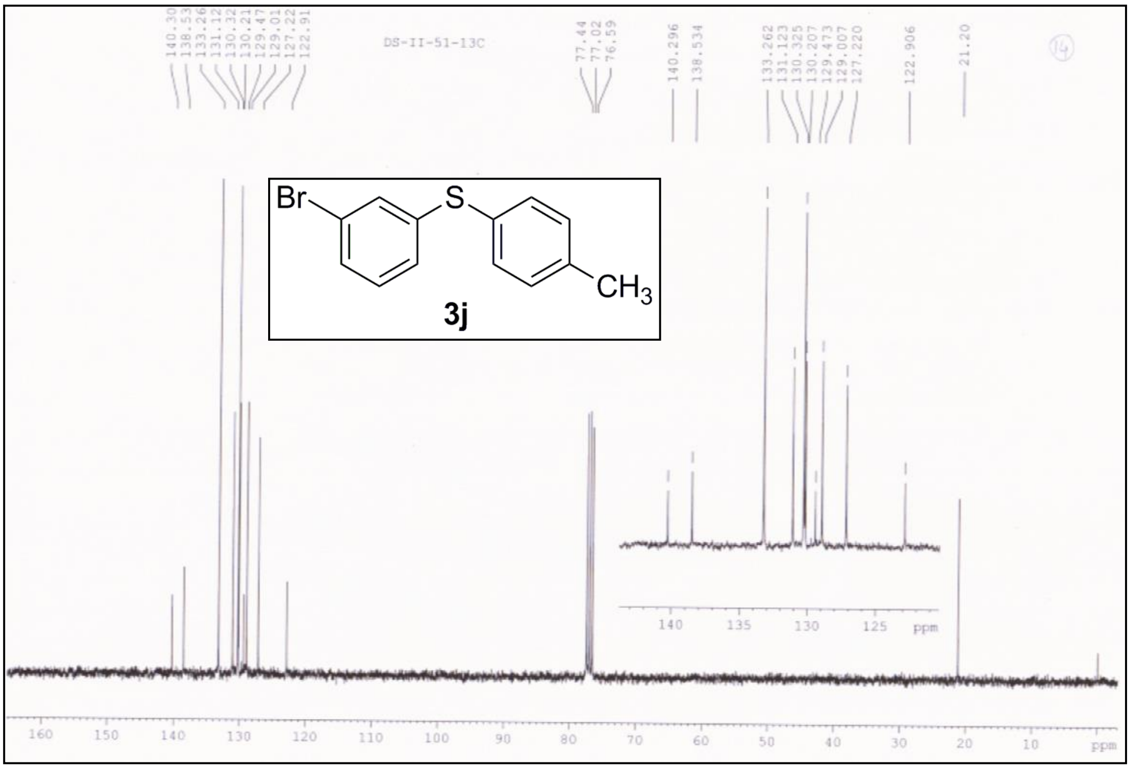

Table 2; entry 11

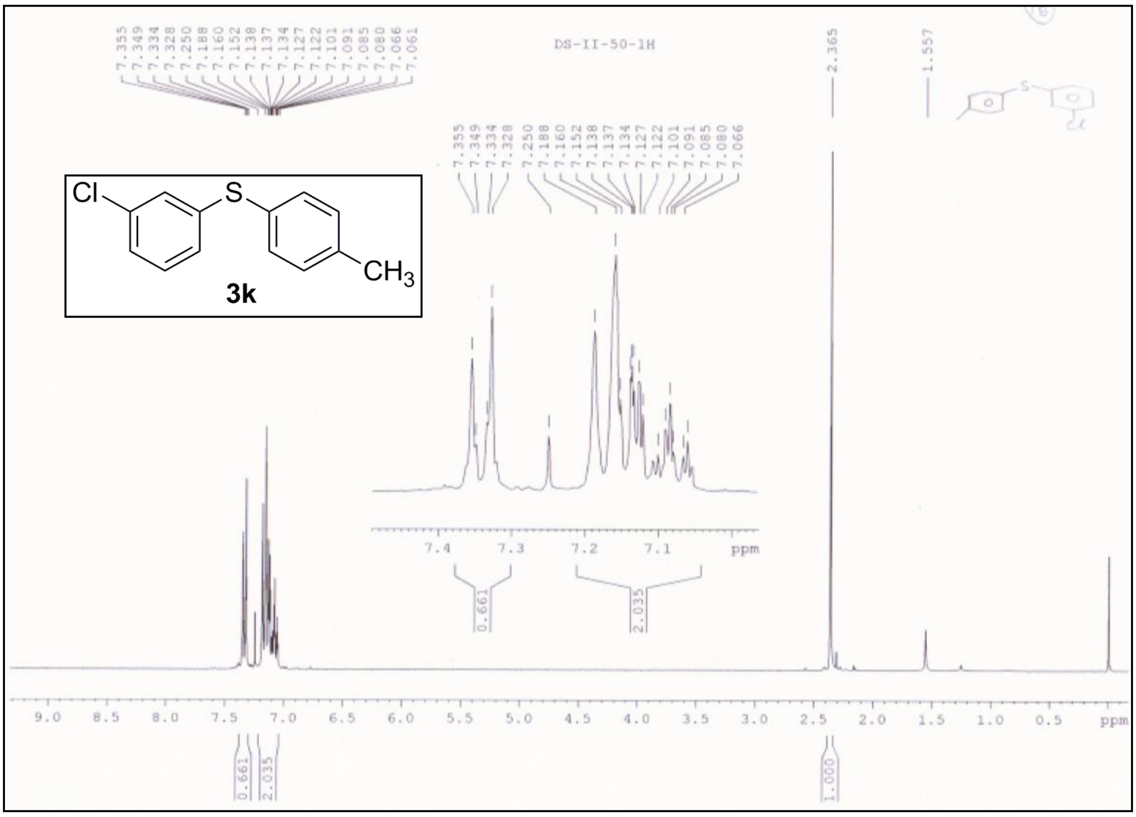

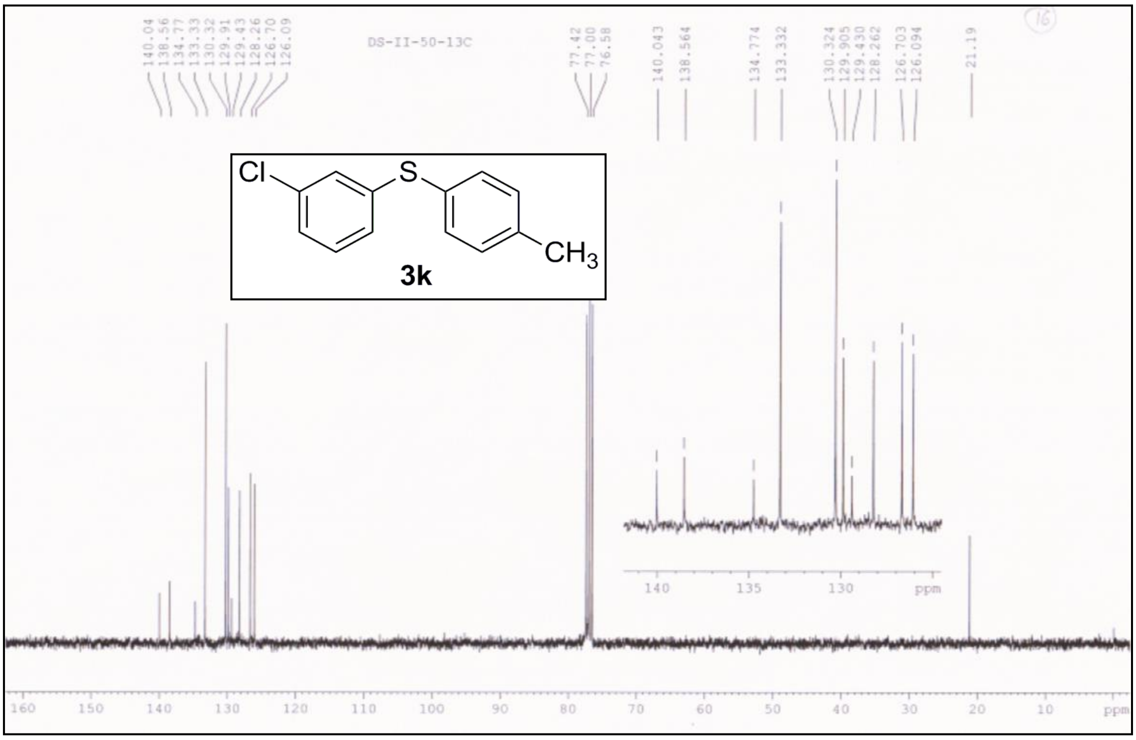

Table 2; entry 12

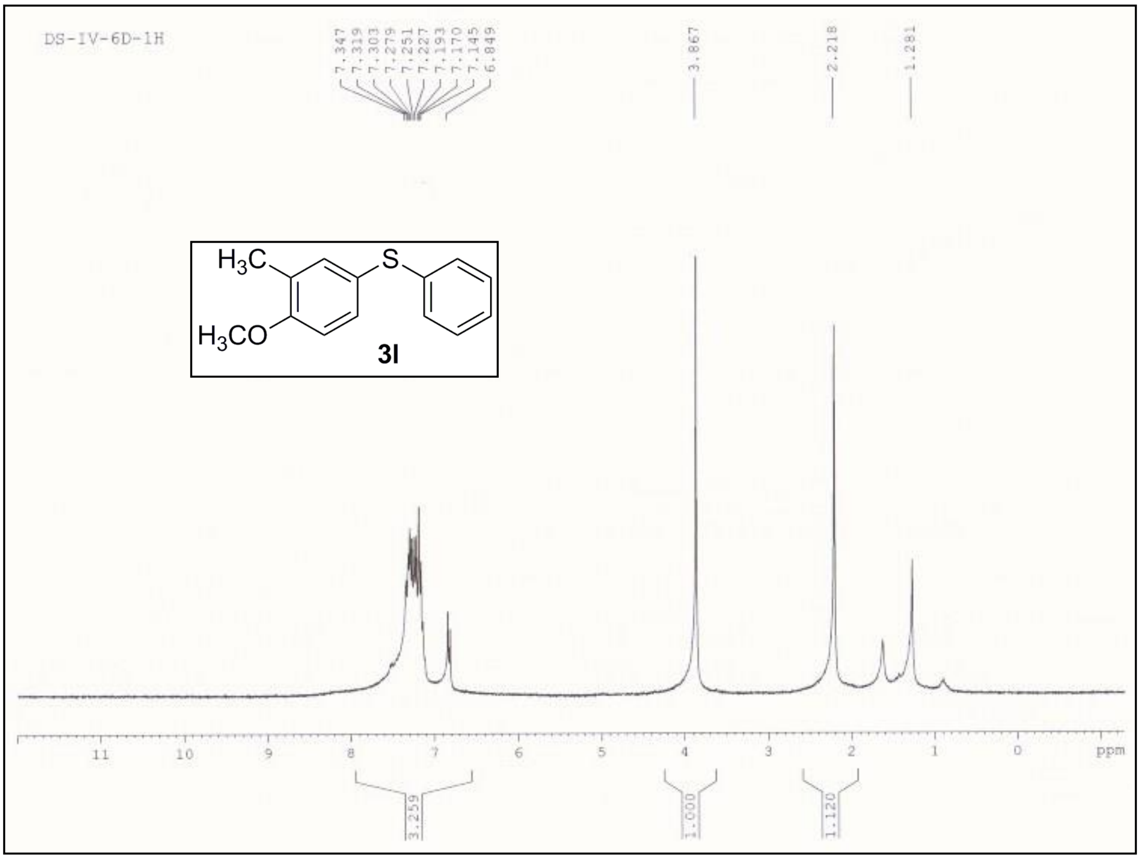

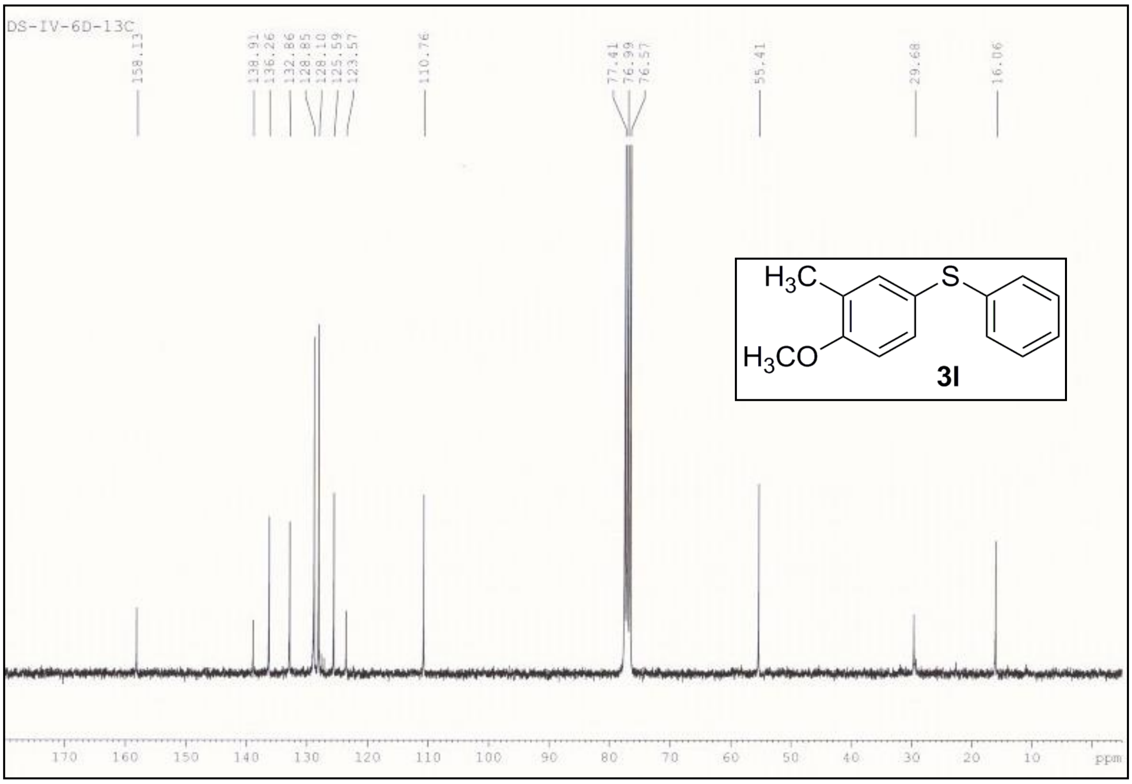

Table 2; entry 14

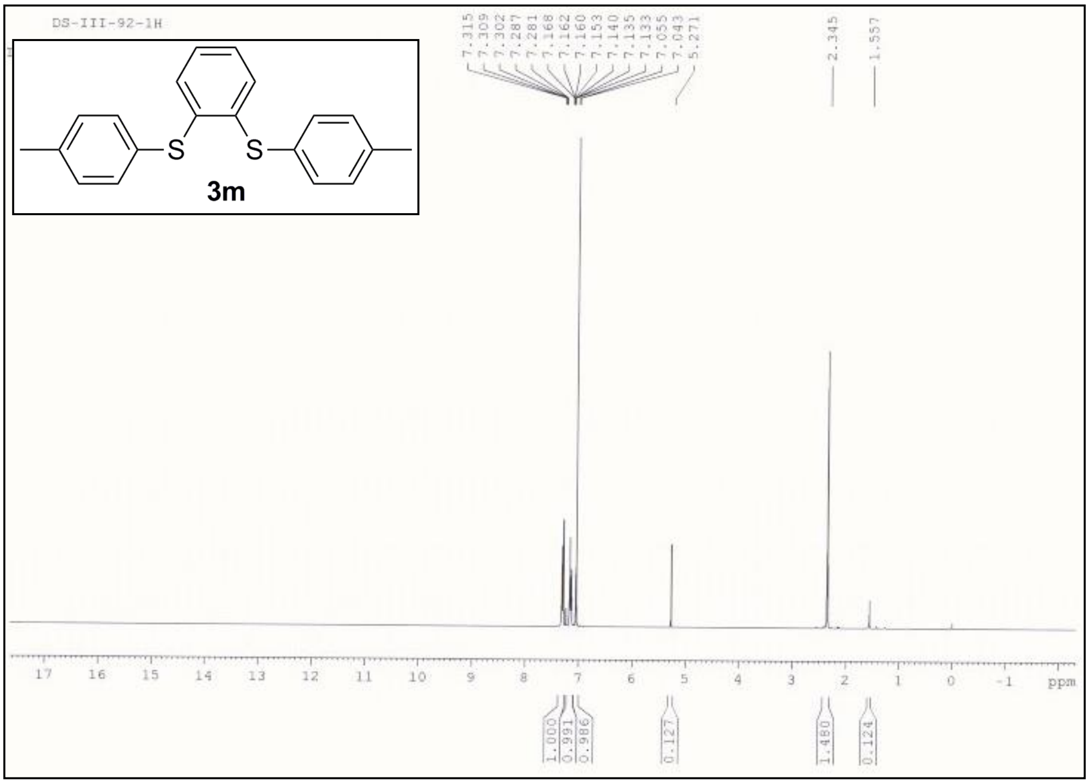

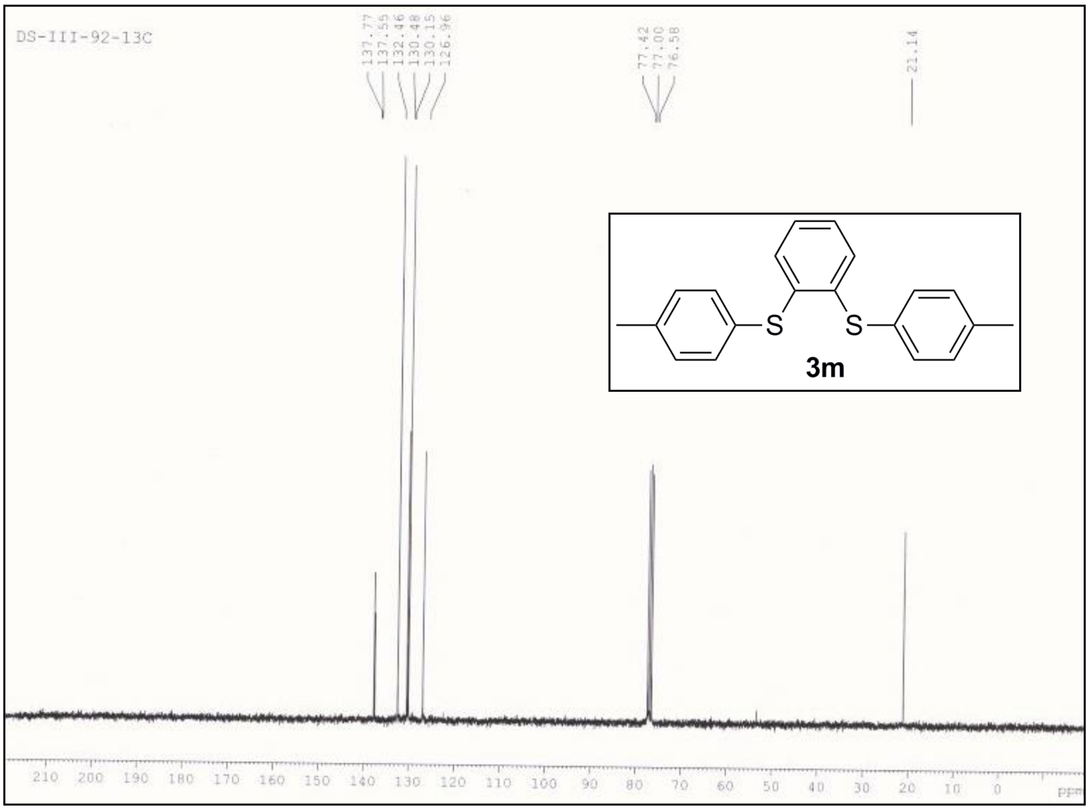

Table 2; entry 15

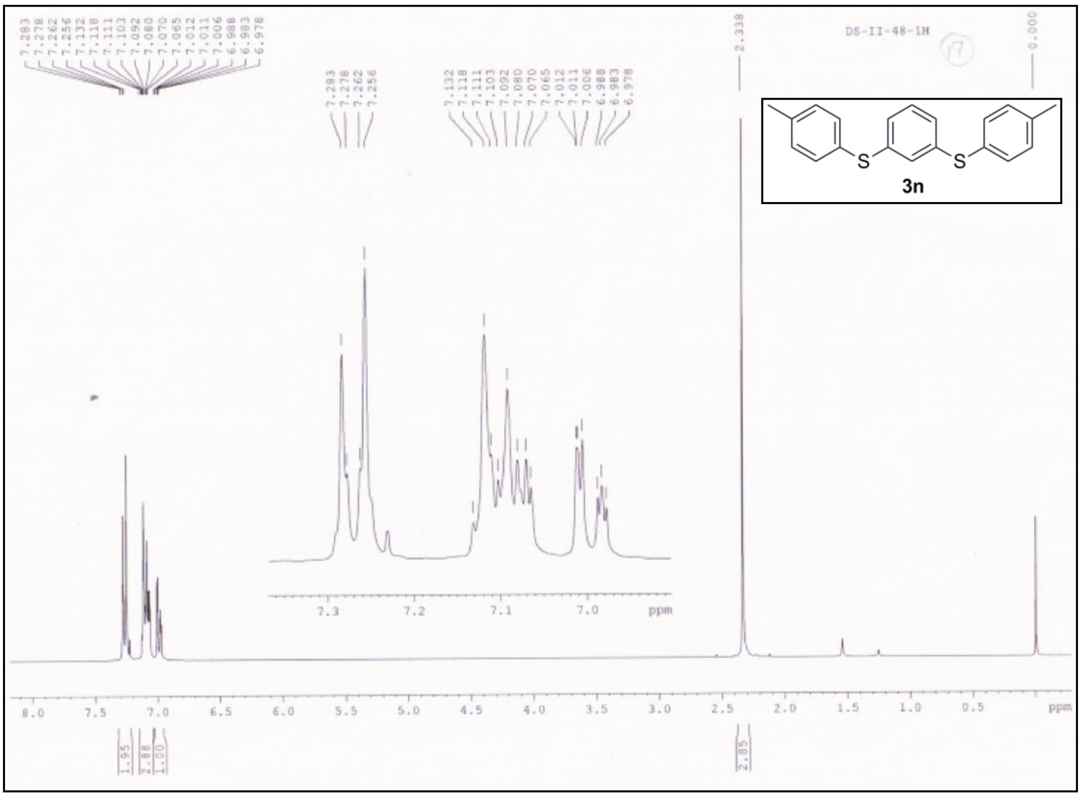

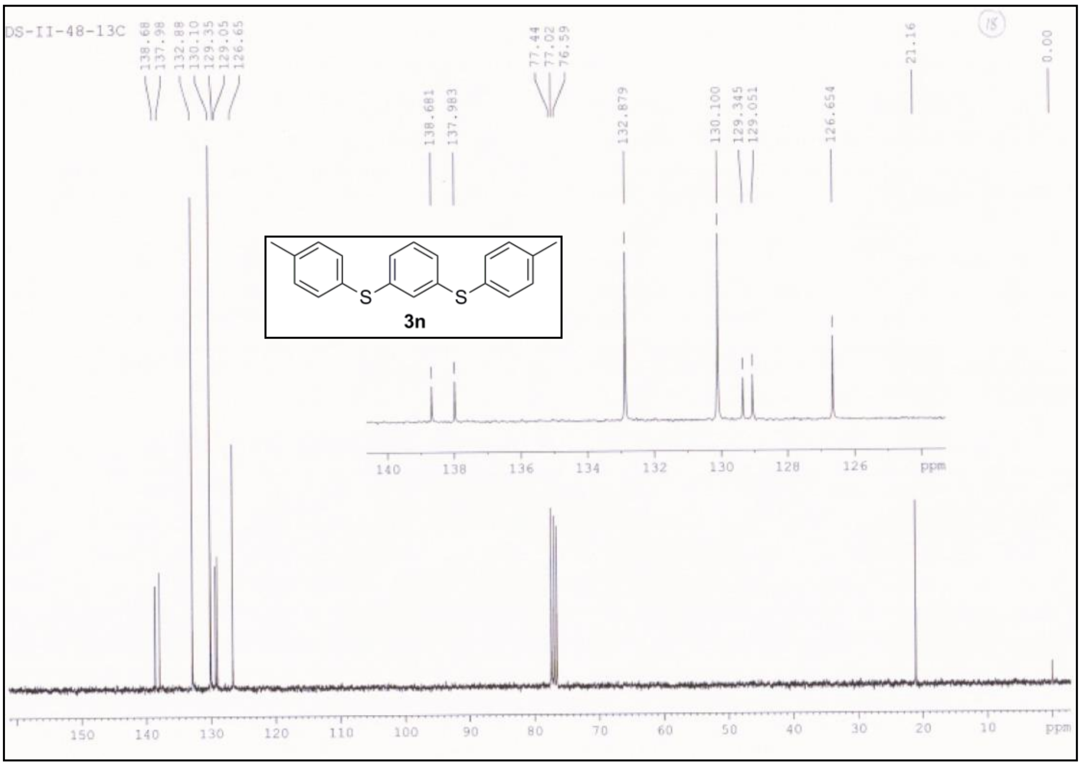

Table 2; entry 16

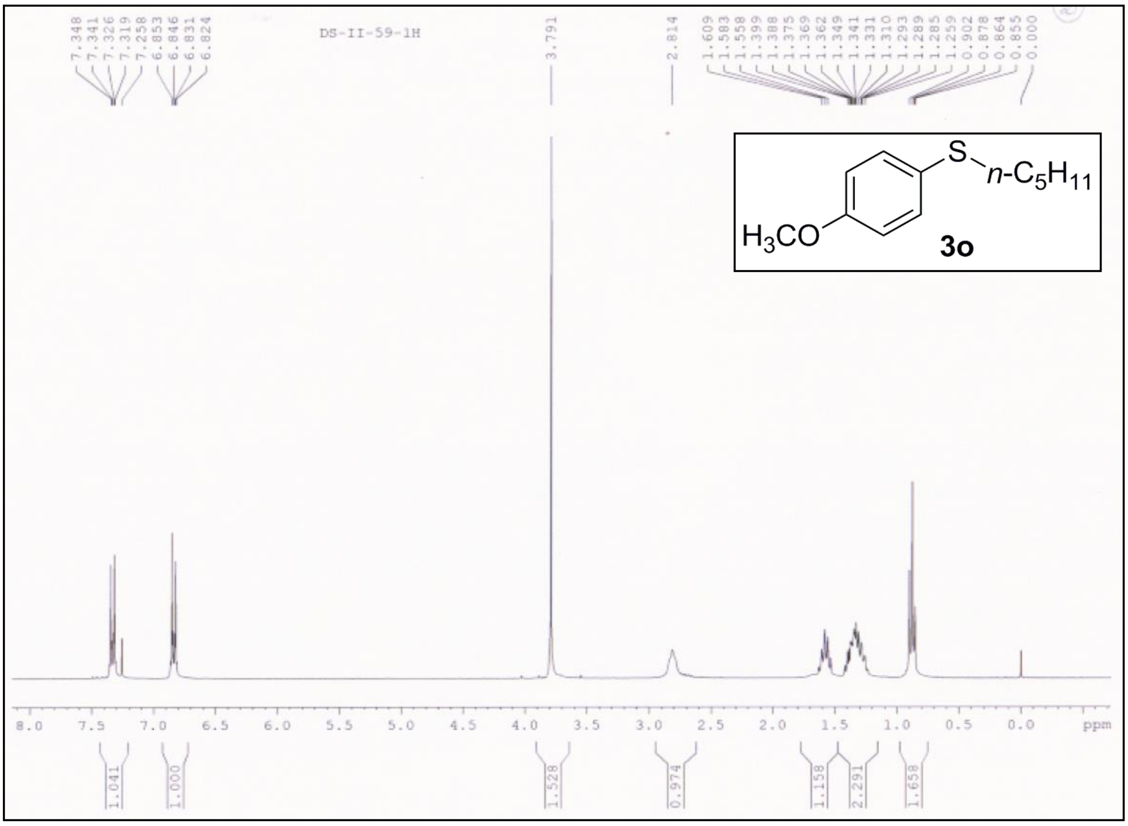

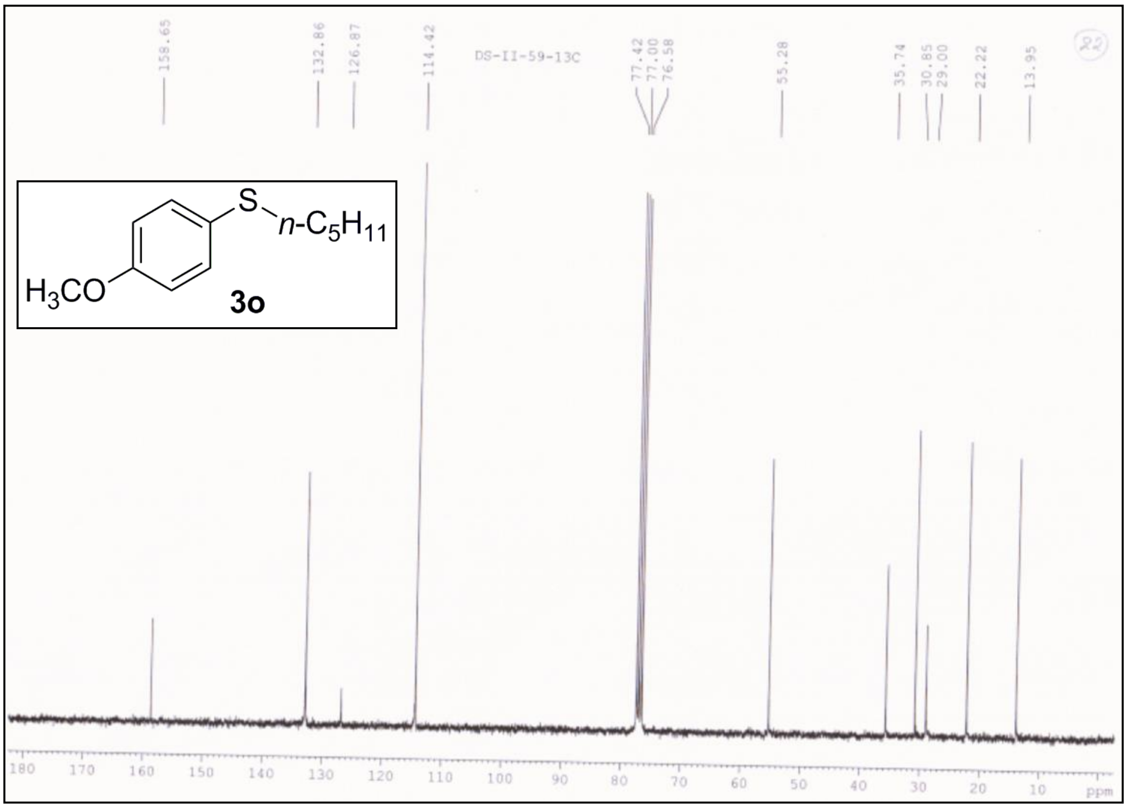

Table 2; entry 17

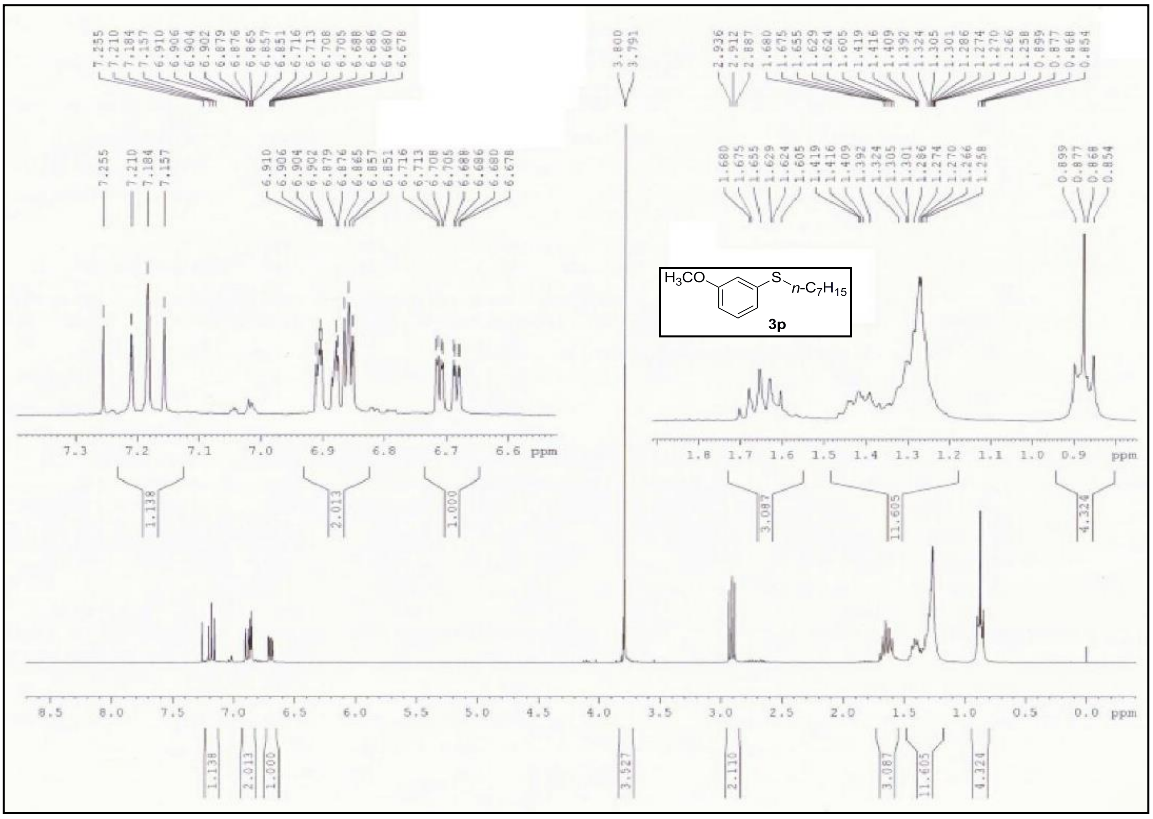

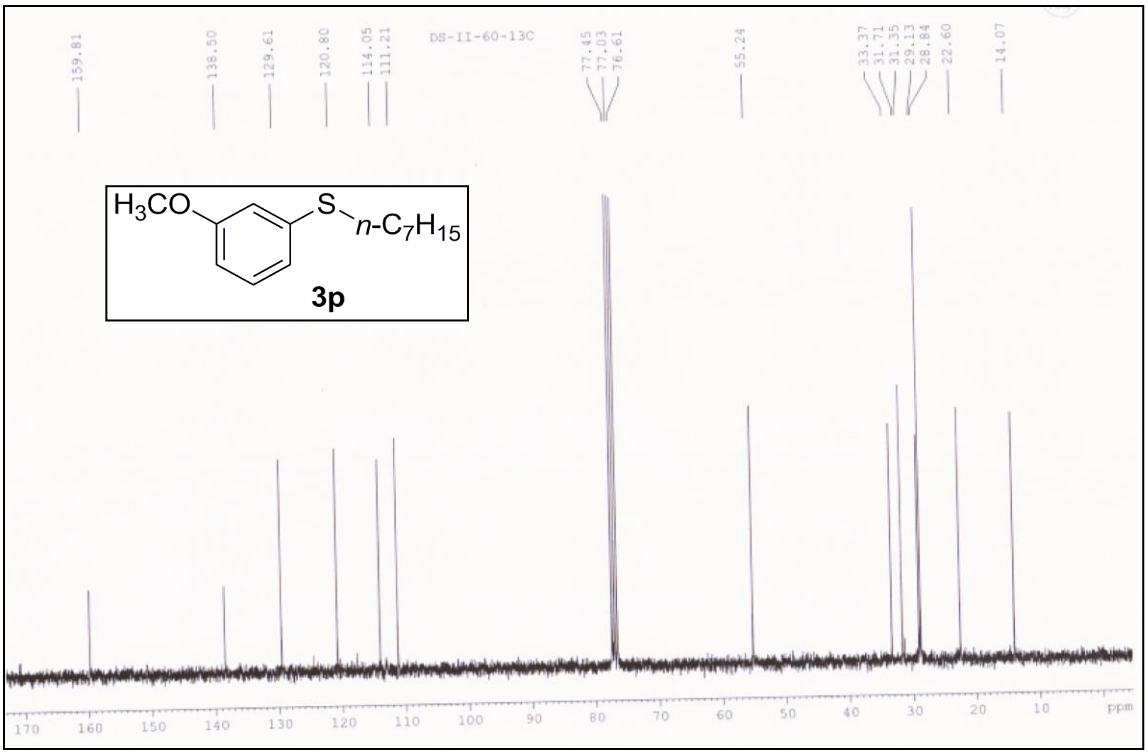

Supplement: File 2 — 1H and 13C NMR spectra (scanned) for compounds 3a–p. [file Beilstein_J_Org_Chem-13-1796-s002.pdf]
